# COVID-19 pandemic experiences of parents caring for children with oesophageal atresia/tracheo-oesophageal fistula

**DOI:** 10.1136/bmjpo-2021-001077

**Published:** 2021-05-18

**Authors:** Alexandra Stewart, Christina H Smith, Simon Eaton, Paolo De Coppi, Jo Wray

**Affiliations:** 1 Department of Speech and Language Therapy, Great Ormond Street Hospital For Children NHS Trust, London, UK; 2 Department of Language and Cognition, University College London, London, UK; 3 Stem Cells and Regenerative Medicine Section, University College London Institute of Child Health, London, UK; 4 Specialist Paediatric and Neonatal Surgery, Great Ormond Street Hospital For Children NHS Trust, London, UK; 5 Centre for Outcomes and Experience Research in Children's Health, Illness and Disability (ORCHID), Great Ormond Street Hospital For Children NHS Trust, London, UK

**Keywords:** COVID-19, qualitative research

## Abstract

**Purpose:**

The COVID-19 pandemic has resulted in a global health crisis of unparalleled magnitude. The direct risk to the health of children is low. However, disease-containment measures have society-wide impacts. This study explored the pandemic experiences of parents of children with oesophageal atresia/tracheo-oesophageal fistula (OA/TOF) in the UK.

**Design:**

A phenomenological approach underpinned use of an asynchronous online forum method, in collaboration with a patient support group. Data were evaluated using thematic analysis.

**Results:**

The online forum ran between 7 November and 18 December 2020 with 109 participants.

Pandemic experiences were divided into themes relating to healthcare and disease containment. Participants described positive experiences with remote healthcare but identified limitations. Delays and cancellations led to escalation of care to an emergency level, slower developmental progress and feelings of being abandoned by services. Inpatient care was perceived as safe but caring alone was emotionally and practically challenging. Disease containment themes revealed anxiety regarding health risks, ‘collateral’ damage to well-being because of isolation, and an impact on finances and employment. Parents described a transition from worry about direct health risks to concern about the impact of isolation on socialisation and development. A process of risk–benefit analysis led some to transition to a more ‘normal life’, while others continued to isolate. Benefits to their child’s health from isolation were reported.

**Conclusions:**

Parents’ experiences of caring for a child with OA/TOF during the pandemic were varied. Rapid adoption of telehealth has demonstrated the enormous potential of remote healthcare delivery but requires refinement to meet the needs of the individual. Future pandemic planning should aim to retain community healthcare services to avoid escalation of care to an emergency, manage chronic and developmental concerns, and support parental well-being. Accurate and consistent disease-specific information is highly valued by parents. Third sector organisations are ideally positioned to support this.

**What is known about the subject?**

- Direct health risk to children from COVID-19 is low but there is a high risk of ‘collateral’ damage from strategies required to contain the virus.
- Patient support groups can be powerful allies in providing accurate and consistent messages, that is particularly useful to those with rare diseases.
- Social media can facilitate rapid and effective data collection in a rare disease cohort.

**What this study adds?**

- A parent perspective of the impact of pandemic-related reduction in healthcare provision and use of telehealth, highlighting areas of need for pandemic and post-pandemic service delivery.
- An insight into parents’ experiences and decision-making surrounding disease-containment measures which highlights the variation, even within a single, rare disease.
- Isolation has resulted in exceptionally low exposure to usual childhood infections resulting in improved health for children with oesophageal atresia/tracheo-oesophageal fistula.

## Introduction

The emergence of SARS-CoV-2 brought about the largest global health crisis for a generation.

Although it is now suggested that children are approximately 50% less likely to be infected than adults, account for 1%–5% of cases worldwide^1 2^ and rarely experience severe disease,^3^ early data indicated that ‘high-risk’ groups for severe disease existed.^4^ As a result, children deemed ‘extremely clinically vulnerable’ were advised to ‘shield’, avoiding all contact with others to minimise their risk of being infected.

One group of vulnerable children are those born with oesophageal atresia/tracheo-oesophageal fistula (OA/TOF). A rare, congenital abnormality, OA/TOF occurs in approximately 1/3500 live births in the UK that results in a blind-ending oesophageal pouch and/or an anomalous connection between the trachea and oesophagus. While survival rates following surgical repair are excellent, many children experience long-term health challenges^5^: swallow dysfunction and feeding difficulties in approximately 80%,^6 7^ gastro-oesophageal reflux and oesophagitis in up to 70%,^5^ recurrent respiratory infections and chronic cough in 40%–52%.^6 8^ Approximately 50% of children have other congenital abnormalities, most commonly cardiac abnormalities.^5^ Hospital readmission with respiratory or gastroenterological issues in the preschool years is common.^9^

Despite vulnerability to respiratory infection, children with OA/TOF have not experienced severe COVID-19 symptoms.^10^ However, disease containment continues to involve restrictions to social contact, education and non-essential business and impacts society beyond the immediate risk to health.^11^ Our aim was to describe parental lived experiences of caring for a child with OA/TOF during the COVID-19 pandemic with the following specific objectives:

1. To describe experiences of accessing healthcare and medical advice.
2. To describe parental experiences of disease-containment measures and their impact.
3. To learn from their experiences and make recommendations for delivery of care for this rare disease.

## Method

A phenomenological approach underpinned use of an online forum to explore parental experiences of accessing healthcare and the impact of disease-containment measures in the UK during the COVID-19 pandemic.^12^

Data were collected using a previously described online forum method and detailed in the online supplemental material.^13^

10.1136/bmjpo-2021-001077.supp1 Supplementary data

In collaboration with TOFS, the UK support group for OA/TOF, a research-specific, private Facebook group was launched. An experienced member of the TOFS Facebook group, independent of the research team, moderated the forum. This online forum was part of a larger study that was granted ethical approval.

### Patient and public involvement

Patient and public involvement (PPI), through collaboration with TOFS and use of a PPI steering group (including four parents), has been integral to study design, recruitment, data analysis and dissemination. Details are provided throughout the Methods section.

### Participants

Convenience sampling was used to recruit parents of children aged 0–18 years with OA/TOF living in the UK. The TOFS support group advertised participation to their members by email and on their Facebook group. Interested parents were asked to apply to join the research Facebook group, with access granted by the moderator after participants consented to participation by agreeing the group ‘rules’; acknowledging responses would be anonymised and passed to the research team. Participants provided demographic data via a link to a separate Survey Monkey questionnaire. These data were used to describe group characteristics but were not linked to individual responses.

### Data collection

Questions for the online forum, provided in box 1, were co-developed with the PPI group and checked by TOFS, ensuring pertinent issues were explored in a sensitive manner. Questions were posted individually by the moderator and participants responded by posting a ‘comment’. Data saturation was assumed once no further comments were being made. A new question was then posted. The moderator answered participant questions, prompted for clarification and invited further responses if required. Participants were able to respond to others’ comments. Participants were also able to respond privately to the moderator rather than posting to the whole group. To diversify participation, parents not using Facebook, or not wishing to share information on the forum, could participate via email.

Box 1 **Online forum questions**

1. If you were expecting any healthcare appointments during the COVID-19 pandemic, what changes or disruptions have you experienced to your/your child’s normal hospital or community care? Follow-up questions
  - Are you concerned about the impact of any changes on your health/the health of your child/the person you care for?
  - What did the services do well under the circumstances?
  - What did not work well?
2. Thinking back to earlier this year, what were you worried about at the beginning of the pandemic? Has this changed over the last 6 months?
3. Were you advised to shield? If so, do you feel that this has had an impact on your child or family?
4. What was your experience of seeking support or advice during the pandemic for OA/TOF-related concerns? Who provided you with the most useful information?
5. How have any OA/TOF-related difficulties impacted on your child’s transitioned back into nursery/childminder/school/college/university, during the pandemic? Please think about the impact on you as a parent and the impact on your child. How have you managed these?
6. During the first wave of the pandemic, did your child’s health impact on your ability to work? Are there ongoing challenges relating to your ability to work?

OA/TOF, oesophageal atresia/tracheo-oesophageal fistula.

### Data analysis

All responses were anonymised by the moderator and sent to the research team as a Word document. Thematic analysis was conducted.^14^ Data were independently coded by three members of the research team, followed by group discussion to agree themes. Two thematic maps were generated. Tables of codes with supporting quotes and the maps were reviewed by the PPI group and two other members of the research team providing data triangulation from different professional and personal perspectives.

## Results

The online forum ran from 7 November to 18 December 2020. There were 109 members, of whom 65 completed the demographic survey (table 1) and responded to at least one question. An additional six participants responded by email.

**Table 1:** Demographic data

Relationship to the child	
Mother	58 (89%)
Father	3 (5%)
Adult with OA/TOF	1 (2%)
Did not respond	3 (5%)
Ethnicity	
White	61 (94%)
Asian or British Asian	1 (2%)
Did not respond	3 (5%)
Geographical location	
England	55 (82%)
Scotland	10 (15%)
Wales	2 (3%)
Age of the child	
Under 2 years of age	25 (38%)
2–4 years of age	33 (34%)
5–11 years of age	12 (18%)
Over 12 years of age	3 (5%)
Type of OA/TOF	
OA and TOF repaired within a week of birth	55 (85%)
OA and TOF repaired more than a week after birth	5 (7%)
OA only	3 (5%)
TOF only	2 (3%)

OA/TOF, oesophageal atresia/tracheo-oesophageal fistula.

### Experiences of accessing healthcare

**Figure 1:**
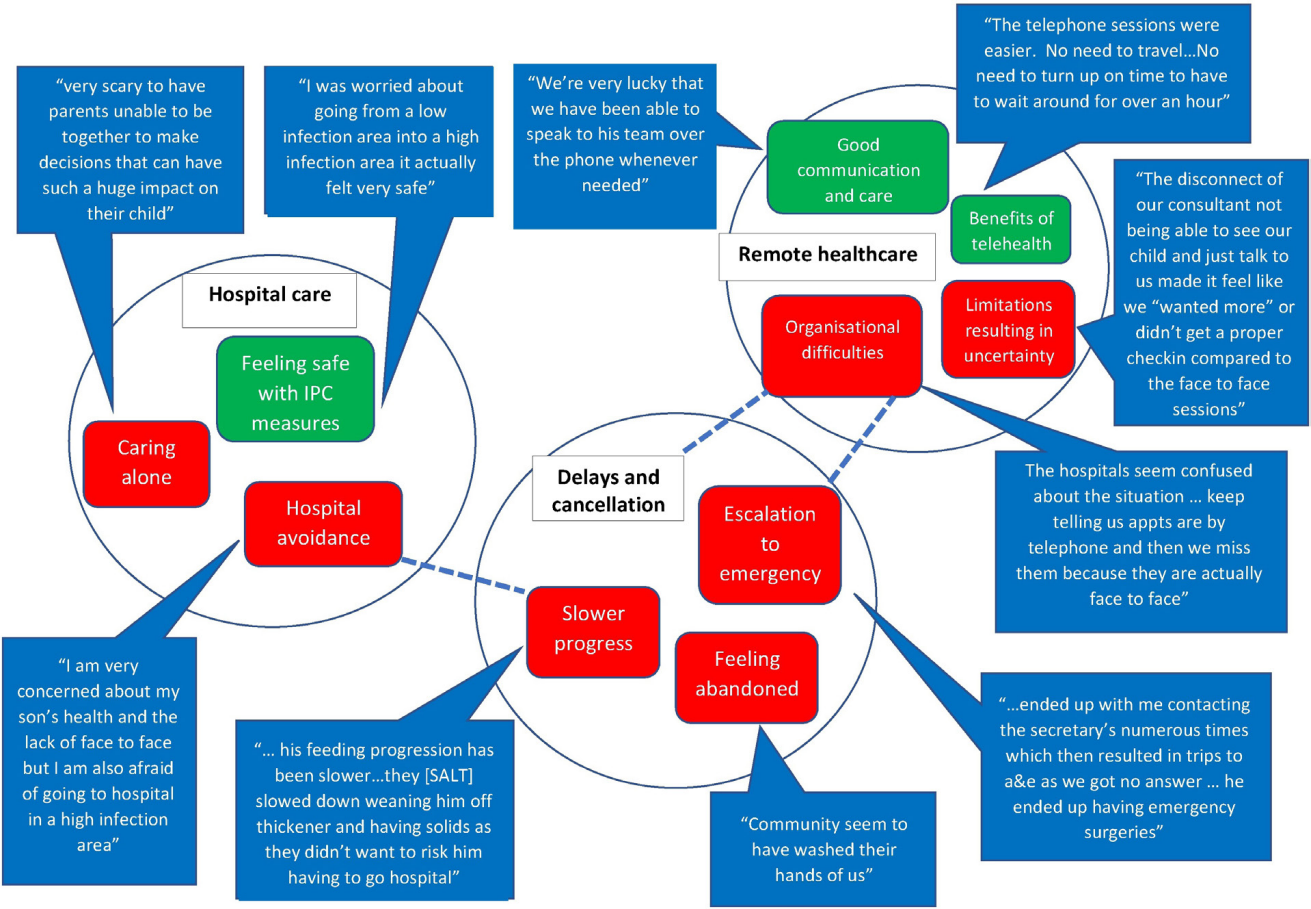
Thematic map healthcare. a&e, Accident and Emergency; IPC, infection prevention and control.

Participants’ experiences of accessing healthcare are summarised in figure 1. Further illustrative quotes are shown in table 2. Themes were grouped into remote healthcare, delays and cancellations, and hospital care.

**Table 2:** Further illustrative quotes relating to healthcare themes

Remote healthcare	Benefits of telehealth	They switched it to a telephone appointment. I was really impressed! The consultant was clearly liking the format too, because he suggested the same next year! Oh, and we were ‘seen’ early - no hanging around for an hour in Clinic 6. It was actually a much better experience than normal.
	Good communication and care	We feel we’ve had good care and lots of support and all our questions answered as we could write them in an email.
		Still a good level of care and compassion even though over the phone.
	Limitations resulting in uncertainty	There appears to have been a real failure to assess the risk of face to face appointments vs the risk to patients of not being seen… had we had a face to face assessment earlier, we might have avoided the blue spells in April/May.
		I am concerned that her health has deteriorated slightly over the last month of two and I really would have liked next week’s TOF clinic to be face to face as I think the respiratory consultant needs to listen to her chest.
	Organisational difficulties	The consultant phoned a couple of weeks before the expected appointment. This meant we weren’t prepared so didn’t ask all the questions/mention things we would have wanted to.
		It’s been very difficult to get advice about health issues while the pandemic has been happening it’s as if my child’s care completely stopped at one point.
Delays and cancellations	Slower progress	We were very concerned about delays to her treatment, and the placing on hold of treatment and check ups, and that the longer term welfare of …. children in particular, was being jeopardised.
		Not having face to face it seems a very very slow process to get him weaned off the tube and start him on solids with the help of SaLT.
	Escalation to emergency	We had to wait months for an elective scope and dilatation, this became an emergency procedure as was not carried out on time and symptoms persisted.
	Feeling abandoned	The only thing I think has been compromised is support from Speech & Language. They seem to have forgotten us & haven't been very helpful
		On many occasions, I would have taken my child in to see the health visitors had they been open.
Inpatient care	Caring alone	…the one parent for hospital stays is incredibly hard. To expect parents not to be with babies when they go in for surgery is really harsh.
		When you have a child to care for, then the ‘one parent’ rule means you’re trying to do multiple jobs at the same time, which is not efficient for the medics, and not good for ensuring your child gets the care needed.
	Hospital avoidance	…advised to be very cautious and during first lockdown we were told by his consultant she didn’t want him anywhere near a hospital (for procedures) as too high risk.
	Feeling safe with infection control measures	We had a heart scan at (hospital name) and that was also mid first lockdown and also very safe and clean. Have no complaints at all!

TOF, tracheo-oesophageal fistula.

#### Remote healthcare

Access to healthcare changed, with a shift to telehealth (telephone or video) appointments reported by most participants.

Benefits to telehealth were identified by many participants: no waiting times or travel, not needing to take time off work and not attending busy waiting rooms. Some parents experienced good, and even improved, communication with healthcare professionals, having telephone or email contact that was not previously available. Where parents felt connected, remote healthcare was positively received. The value of specialist nurses in achieving good communication was evident.

We’ve had huge support from our CNS team who has been amazing throughout the entire journey.

However, limitations resulting in uncertainty were also described. Some parents raised concerns that their child’s health or development was being compromised. Parents reported feeling disconnected from their healthcare team, due to communication or organisational challenges and the limitations of telehealth appointments. A few expressed concern at how their child would cope with face-to-face appointments after a period of not attending in person.

I’m worried that my daughter will become withdrawn, nervous, anxious for future appointments.

#### Delays and cancellations

Delays and cancellations to inpatient and outpatient care were widely reported. Most cancelled appointments had been rebooked.

Participants described concern at anticipated and realised difficulty accessing timely and appropriate care, with stark accounts of feeling abandoned by healthcare services. This was most evident for community services, with speech and language therapy services most frequently cited.

Parents felt that delays impacted directly on their child’s health, including escalation of care to an emergency and slower developmental progress.

#### Hospital care

Hospital avoidance due to concerns about infection risk was reported but all accounts of hospital treatment were positive. Parents felt safe with infection prevention and control measures.

Caring alone, due to one parent policies, caused the greatest challenge. Participants described distress making decisions regarding care, including surgery alone, the absent parent being omitted from care and the challenge of processing information while simultaneously looking after the child.

One parent highlighted the impact of mask-wearing on bonding. Practical challenges, such as not having a parent kitchen and car-parking, were also reported. Overall, access to hospital care was reported more positively than community care.

### Experiences of disease containment and their impacts

**Figure 2:**
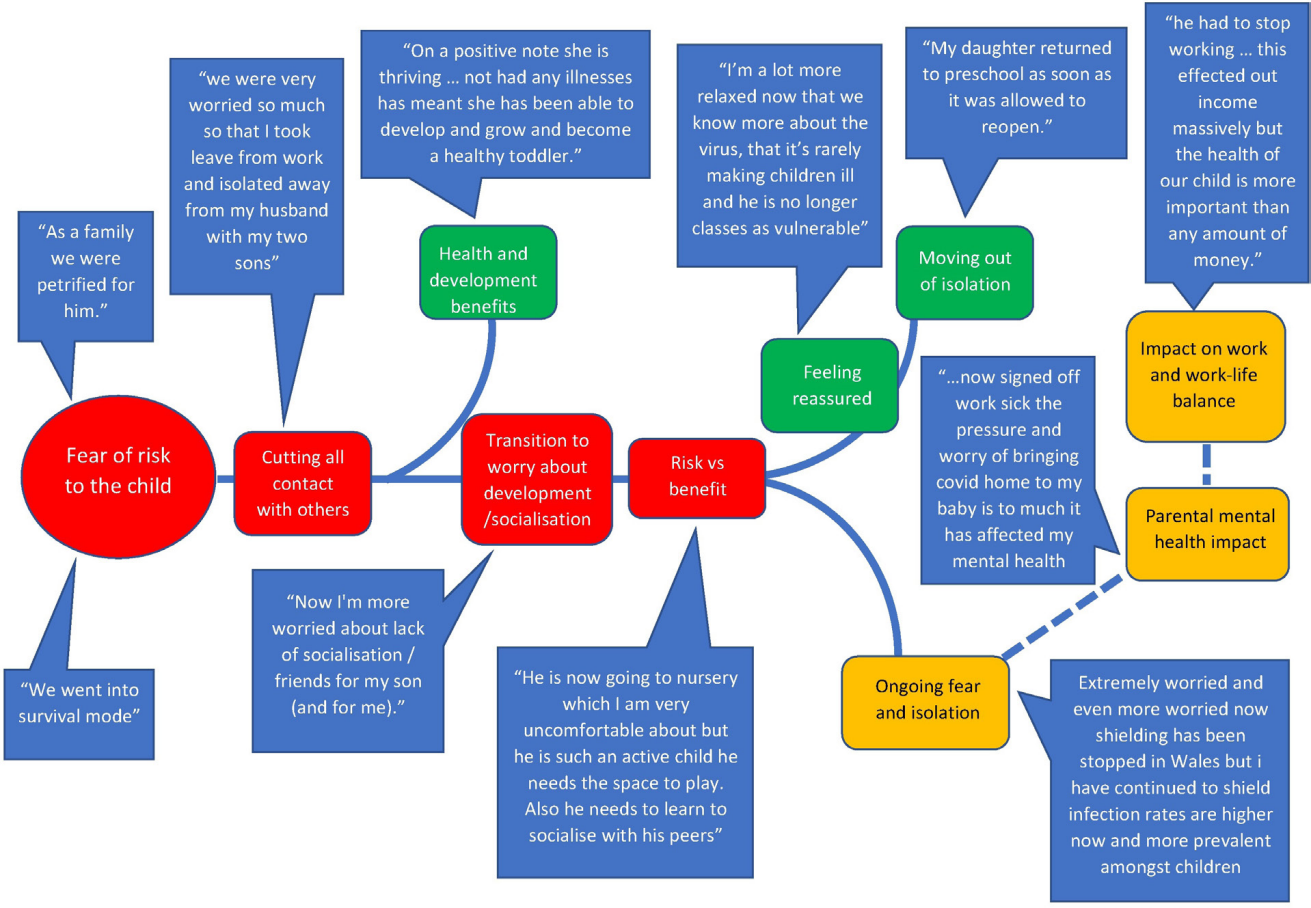
Thematic map non-healthcare.

Themes relating to disease containment are outlined in figure 2, with further illustrative quotes provided in table 3.

**Table 3:** Further illustrative quotes relating to disease containment

Fear of risk of the child	In the beginning we were extremely concerned and worried about our son catching the virus as months before we had been In hospital for just a cold.
	At the beginning, I think like most people, seeing people on ventilators with respiratory issues was extra concerning for TOFs.
Cutting all contact with others	It was extremely stressful, we completely cut off contact with friends and family and shielded, which was difficult and upsetting.
	We completely shielded too to be safe so none of us at home left the house (apart from me walking the dog) March to August.
Transition to worry about socialisation/development	Now my main worry is him getting the care and support he needs to develop during the crisis.
Risk versus benefit	My son is 3 and I personally think he needs to social interaction with other kids and family members. It’s a risk- but keeping him cooped up is not natural.
Feeling reassured	I was relieved when his respiratory consultant explained he no longer needed to shield.
	TOFS was ever a great source of support and information by direct posts of latest information and from other parents and their experiences. My son’s two consultants were very helpful in putting my mind at rest too.
Moving out of isolation	Now we are back to normal and he is going to nursery we are as careful as possible in terms of hygiene but are living as normal now.
	It was a worry with our little boy starting Reception… It did ease our minds a little to know the school was doing everything they could.
Ongoing fear and isolation	I’m not sure if he should be in school or not but I’m keeping him home.
	We weren’t advised to shield but did anyway until August and remain cautious. Our TOF is 10 months now - his grandparents have only held him twice and he has yet to meet Uncles and cousins.
Parental mental health impact	I had finally (after poor care at the outset meant we were never seen by the counsellor) started getting help for PTSD.
	For the last 6 weeks I have been signed off because the pressure overwhelmed me. I have been signed off for a further month and referred for counseling by HR.
Impact on work and work–life balance	My husband’s ability to work was affected since he was at home with a screaming child usually with him (I was entertaining the 2 year old).
	More work, less time, more pressure to do housework and make the meals due to being at home - and having to create time to make sure we were getting lots of exercise- all his health needs are managed by me.

HR, human resources; PTSD, post-traumatic stress disorder; TOF, tracheo-oesophageal fistula.

#### Fear of risk to the child and cutting contact

Fears for their child’s health were expressed almost universally. Several parents described an overwhelming fear that their child was going to die.

I couldn’t shift the feeling again that we that we were going to lose him.

This led to most participants cutting all contact with others outside their household; some following the UK government’s advice to ‘shield’, others without this advice. Whether and when children were advised to shield varied. Some received instruction at the start of the pandemic, some after a couple of months and some not at all. Where shielding advice was not immediate, many participants sought information from healthcare teams and the TOFS support group. Participants identified the TOFS website and online peer support as particularly helpful.

The TOFS Facebook group is the only place where I have seen useful information about our TOFS.

Shielding experiences were varied. Some felt gratitude for the family time. For others, balancing work with children at home or the social isolation resulted in high levels of stress.

#### Transitioning to new worries

Many parents transitioned from worrying about their child’s health to worrying about socialisation and development. They described balancing the health benefits of isolation with the risks to well-being.

Participants highlighted the burden of assessing the risks/benefits of school or childcare attendance. Good communication with the school/nursery and trust in the infection control procedures facilitated attendance. Some reported that infection control measures prevented in-person staff training, disrupting transition to school/nursery. One child was unable to access longer hours in nursery as the parent was unable to go on-site to gastrostomy-feed her child. Two parents highlighted to school staff their child’s chronic OA/TOF-related cough to differentiate it from an infectious COVID-19-related cough.

…wanted to explain that her TOF cough was normal for her…It wasn’t ideal having to try to explain at a distance at the door, but I didn’t want her new teacher to be alarmed (or other kids in the class) when she coughed.

#### Moving out of isolation and ongoing fear and isolation

Fear led to complete isolation for almost all participants initially, but as the pandemic progressed experiences diverged. For some, increased knowledge provided reassurance. This, coupled with increasing concern about the social and emotional effects of isolation, prompted transition to a more ‘normal life’, although within society-wide restrictions. For others, increased information and knowledge was not reassuring, resulting in continued isolation and anxiety, including continued home-learning.

#### Health and development benefits

Strikingly, many parents reported benefits of social isolation, highlighting reduced illness and hospitalisations, improved weight gain and improvements to general development.

#### Impact on parental mental health and work–life balance

Parents made direct reference to the impact that isolation as a result of disease-containment measures, difficulty accessing healthcare and anxiety about the health risk to their child had on their own mental health. A small number required professional support for anxiety or post-traumatic stress disorder.

Combining childcare with home working, managing with limited space and an increased burden of domestic tasks were stress-inducing. Financial hardship was reported. The ability to work was affected by the need to look after children, avoidance of social contact to keep their child safe and parental mental health difficulties.

## Discussion

The online forum allowed for timely gathering of parental insights into the impact of the COVID-19 pandemic on children with OA/TOF. The method engaged a large number of parents from a rare disease cohort, avoided face-to-face contact and minimised burden by allowing for asynchronous participation.

Access to healthcare during the pandemic has been shaped by infection prevention and control measures, limiting face-to-face contact and prioritisation of ‘essential’ services. This study highlights the significant impact these changes have had.

Telehealth was widely and rapidly adopted. No parents reported having accessed telehealth prior to the pandemic. Benefits, including access to specialist services from geographically distant locations and reduced costs and time to patients reported in this study, have long been recognised.^15^ Recent technological advances in mobile communication, software and high-speed internet have increased feasibility of telehealth^15^ and effective use in paediatric surgical conditions has been demonstrated.^16 17^

While many parents reported receiving good care remotely, wanting this to continue post-pandemic, some felt disconnected from their healthcare team and that care was suboptimal. These views are echoed by clinicians.^18^ Our study design prevented linking demographic data to individual responses. However, diagnostic complexity and the age of the child varied within the group. Younger children with OA/TOF tend to have more challenging health needs with postoperative morbidity greater for some.^9 19^ We propose satisfaction with telehealth was greater for those at a stable point in their care than those with specific concerns. Dissatisfaction was not reported in other pre-pandemic studies evaluating use of telehealth with paediatric surgical patients.^16 20^ This may be due to the speed with which telehealth was rolled out, with blanket, rather than targeted, use.

It is likely that use of telehealth will continue post-pandemic. Implementation of telehealth long term will be reliant on development of pathways enabling a balance of face-to-face and remote appointments with an analysis of the impact on care.^21^ Bird and colleagues^22^ have developed a comprehensive co-design framework to develop virtual clinics for the management of chronic illness in children. Use of such a framework would ensure feelings of disconnect identified in this study are minimised. Service providers must now actively engage with the rapidly evolving technology, such as use of remote stethoscopes,^23^ understand barriers and support access to technology,^21^ and develop appointment-specific telehealth guidance to optimise use of telehealth.^24^

Parents described the impact that delays and cancellations had to their child’s health and developmental progress, with access to community care particularly problematic. A number of parents highlighted the distress caused by feelings of being abandoned by healthcare services. Redeployment had a significant impact on services identified as ‘non-essential’.^25^ Our findings mirror those of research with other rare diseases.^26^ Continuation of ‘non-essential’ services, such as community speech and language therapy, during periods of high resource would reduce the need for escalation of care to emergency levels, promote development and support parental well-being.

The wider harms caused by society-wide ‘lockdown’ have been well summarised^11^ and are reflected in our findings; fear and anxiety, displaced non-COVID care, social isolation, stress and loss of income were all reported. Similar themes emerged from research exploring the experiences of parents caring for children with cancer.^27^ Our findings highlight the diversity of experiences, typified by divergent management of anxiety that enabled some to transition to a more normal life, while others continued to isolate. Although individual differences in risk evaluation are inevitable, clinicians should acknowledge the burden of decision-making and seek to support by providing clear communication of the best available evidence to mitigate unnecessary isolation.

Parents highlighted the challenge of obtaining information about risks to their child. Not all children were identified as ‘extremely clinically vulnerable’ and advice to shield was mixed. Mixed messages and the need for parents to make their own decisions increased anxiety, also identified in parents of children with cancer.^27^ Clinical nurse specialists have been shown to improve patient outcomes,^28^ and were identified in this study as excellent sources of advice. The TOFS support group also played an important role in provision of disease-specific information. While individualised assessment may be required, communication between the government and/or healthcare providers and the charitable sector should be leveraged as a means of sharing accurate and consistent information.

Many parents described the positive impact that isolation had on their child’s health and growth. Exposure to common viruses and other infections is usually an inescapable part of childhood and supports development of a well-functioning immune system^29^ but can necessitate hospital treatment for vulnerable children. The long-term impact of not being exposed is unknown. However, clinicians should be aware of the potential challenge that some parents will face in supporting normal childhood activity with the knowledge that avoidance may improve health post-pandemic.

Chronic cough is common in children with OA/TOF.^8 30^ Interestingly, difficulty differentiating infective coughing from the child’s usual cough was not commonly reported and was not hindering school attendance. TOFS provide excellent resources for families to educate about chronic ‘TOF cough’, limiting misunderstanding and empowering parents to advocate appropriately.

Recommendations, based on parental lived experience data, for OA/TOF service providers are presented in table 4. While this study focused solely on children with OA/TOF, it is likely that many of these recommendations would be appropriate for children with other complex healthcare needs.

**Table 4:** Recommendations for practice

Needs identified	Recommendations for OA/TOF service delivery	Applicable to general service delivery	Applicable to pandemic service delivery
Consistent communication, access to information	Provide a single point of contact within specialist multidisciplinary specialist services, for example, clinical nurse specialist	●	●
	Engage with, and signpost to, third sector organisations to deliver disease-specific information	●	●
Optimisation of remote healthcare	Use a co-design framework to develop telehealth services to support individualisation of care and meet patient/parent needs	●	●
	Invest in technology to support assessment at home	●	●
Avoidance of harm	Maintain community healthcare services even during periods of high resource need, wherever possible		●
	Acknowledge the burden of parental decision-making during routine follow-up appointments		●
	Identify parental anxiety/mental health concerns related to child’s health/development, signposting for appropriate support	●	●

OA/TOF, oesophageal atresia/tracheo-oesophageal fistula.

### Limitations

Despite efforts to facilitate wider involvement, participants were overwhelmingly female white parents of preschool children, likely reflecting those most commonly using Facebook and engaging with a support group. We acknowledge that although a wide range of experiences were described, they may not be reflective of the whole OA/TOF community.

Description of group rather than individual demographics supported anonymity but prevented subanalysis by OA/TOF type or age. Future research should identify whether such factors impact on satisfaction with telehealth and the assessment of risk.

## Conclusion

Parents’ experiences of caring for a child with OA/TOF during the pandemic were varied. Rapid adoption of telehealth has demonstrated the enormous potential of remote healthcare delivery but requires refinement to meet the needs of the individual. Future pandemic planning should aim to retain community healthcare services to avoid escalation of care to an emergency, manage chronic and developmental concerns, and support parental well-being. Accurate and consistent, disease-specific information is highly valued by parents. Third sector organisations are ideally positioned to support this.

## Supplementary Material

Author's manuscript

## Data Availability

No data are available. Although data have been anonymised, we have not made data available to protect the identity of those involved in the research, due to the detail provided by participants.
