# Supplementary material for: COVID-19 pandemic experiences of parents caring for children with oesophageal atresia/tracheo-oesophageal fistula
Source: BMJ Paediatr Open. 2021 May 18;5(1):e001077. doi: 10.1136/bmjpo-2021-001077 (PMC8136802; doi:10.1136/bmjpo-2021-001077)
Supplement: Supplementary data [file bmjpo-2021-001077supp001.pdf]

Supplementary material

A step-by-step outline of the online forum method

Time point	Task	Action completed by
Pre-recruitment	Write and agree standard operating procedure with support group/charity	Research team<br>PPI group<br>Support group/charity
	Write participant information sheet including statements regarding data use, data storage, confidentiality	Research team<br>PPI group
	Appoint forum moderator	Research team<br>Support group/charity
	Develop questions	Research team<br>PPI group<br>Support group/charity
	Write recruitment advert/publicity material	Research team
	Create Facebook group: set group rules, screening questions for group membership, privacy settings	Research team<br>Forum moderator
Recruitment	Distribute recruitment advert/publicity material (website, email, social media)	Support group/charity
	Interested participants complete screening questions and consent to participation through agreement to group rules	Participants
	Participants meeting criteria for group accepted	Forum moderator
	Participants not meeting criteria for group declined e.g. healthcare professionals	Forum moderator
Data collection	Participants invited to complete demographic questionnaire via link to Survey Monkey	Forum moderator<br>Participants
	First question posted	Forum moderator
	Participants post response to question as a Facebook "comment". Participants engage with other's comments if they wish using "reply"	Participants
	Prompt for more information, further responses or clarification at the moderator's discretion	Forum moderator
	Responses monitored to ensure adherence to group rules. Inappropriate or offensive posts removed	Forum moderator

	Further questions posted one at a time, once no further responses to previous question (assumed data saturation)	Forum moderator
Data analysis	Responses copied into a Word document and anonymised	Forum moderator
	Anonymised transcript passed to research team	Forum moderator
	Thematic analysis conducted	Research team
	Analysis triangulated with PPI group	Research team<br>PPI group

Group rules that had to be agreed to before being accepted into the group

**Anonymity**<br><br>I understand that I will provide basic personal information to allow the research team to know who took part in the forum but that they will not be able to identify me by name.
**Information sharing**<br><br>I understand that the results of the study will be published and shared with professionals at conferences and in journals. A summary of the results will be available on the TOFS website and shared in the CHEW newsletter.
**Information sharing (2)**<br><br>I understand that information I give in relation to this project in any online posts may be anonymised and directly quoted in publications and presentations.
**Respect everyone's privacy**<br><br>Being part of this group requires mutual trust. Authentic, expressive discussions make groups great, but may also be sensitive and private. I understand that what's shared in the group should stay in the group.
**Provide information sensitively**<br><br>Please do not use your child's name, or the name of any other individual when you are posting. Please do not post photos or other identifiable content.
