# Supplementary material for: COVID-19 pandemic experiences of parents caring for children with oesophageal atresia/tracheo-oesophageal fistula
Source: BMJ Paediatr Open. 2021 May 18;5(1):e001077. doi: 10.1136/bmjpo-2021-001077 (PMC8136802; doi:10.1136/bmjpo-2021-001077)
Supplement: Author's manuscript [file bmjpo-2021-001077.draft_revisions.pdf]

BMJ Paediatrics Open

BMJ Paediatrics Open is committed to open peer review. As part of this commitment we make the peer review history of every article we publish publicly available.

When an article is published we post the peer reviewers' comments and the authors' responses online. We also post the versions of the paper that were used during peer review. These are the versions that the peer review comments apply to.

The versions of the paper that follow are the versions that were submitted during the peer review process. They are not the versions of record or the final published versions. They should not be cited or distributed as the published version of this manuscript.

BMJ Paediatrics Open is an open access journal and the full, final, typeset and author-corrected version of record of the manuscript is available on our site with no access controls, subscription charges or pay-per-view fees (http://bmjpaedsopen.bmj.com).

If you have any questions on BMJ Paediatrics Open's open peer review process please email info.bmjpo@bmj.com

BMJ Paediatrics Open

The impact of COVID-19 on children with oesophageal atresia and/or tracheo-oesophageal fistula (OA/TOF)

Journal:	*BMJ Paediatrics Open*
Manuscript ID	bmjpo-2021-001077
Article Type:	Original research
Date Submitted by the Author:	01-Mar-2021

Complete List of Authors:	Stewart, Alexandra; Great Ormond Street Hospital For Children NHS Trust, Department of Speech and Language Therapy; University College London, Department of Language and Cognition<br>Smith, Christina; University College London, Language and Cognition<br>Eaton, Simon; University College London Institute of Child Health, Paediatric Surgery<br>De Coppi, Paolo; Great Ormond Street Hospital For Children NHS Trust, General Surgery; University College London Institute of Child Health, Developmental Biology and Cancer<br>Wray, Jo; Great Ormond Street Hospital For Children NHS Trust, Centre for Outcomes and Experience Research in Children's Health, Illness and Disability (ORCHID)

Keywords:	COVID-19, Qualitative research

SCHOLARONE™
Manuscripts

I, the Submitting Author has the right to grant and does grant on behalf of all authors of the Work (as defined in the below author licence), an exclusive licence and/or a non-exclusive licence for contributions from authors who are: i) UK Crown employees; ii) where BMJ has agreed a CC-BY licence shall apply, and/or iii) in accordance with the terms applicable for US Federal Government officers or employees acting as part of their official duties; on a worldwide, perpetual, irrevocable, royalty-free basis to BMJ Publishing Group Ltd ("BMJ") its licensees and where the relevant Journal is co-owned by BMJ to the co-owners of the Journal, to publish the Work in this journal and any other BMJ products and to exploit all rights, as set out in our licence.

The Submitting Author accepts and understands that any supply made under these terms is made by BMJ to the Submitting Author unless you are acting as an employee on behalf of your employer or a postgraduate student of an affiliated institution which is paying any applicable article publishing charge ("APC") for Open Access articles. Where the Submitting Author wishes to make the Work available on an Open Access basis (and intends to pay the relevant APC), the terms of reuse of such Open Access shall be governed by a Creative Commons licence – details of these licences and which Creative Commons licence will apply to this Work are set out in our licence referred to above.

Other than as permitted in any relevant BMJ Author's Self Archiving Policies, I confirm this Work has not been accepted for publication elsewhere, is not being considered for publication elsewhere and does not duplicate material already published. I confirm all authors consent to publication of this Work and authorise the granting of this licence.

Confidential: For Review Only

The impact of COVID-19 on children with oesophageal atresia and/or tracheo-oesophageal fistula (OA/TOF)

Stewart, A.[1,2], Smith, C.[2], Eaton, S.[3], De Coppi, P.[3,4] and Wray, J. [5].

Corresponding author: Alexandra Stewart, Department of Speech and Language Therapy, Great Ormond Street Hospital, Great Ormond Street, London, WC1N 3JH.

Email: Alex.stewart@gosh.nhs.uk

1. Department of Speech and Language Therapy, Great Ormond Street Hospital, London, UK
2. Department of Language and Cognition, Division of Psychology and Language Sciences, University College London, London, UK
3. Developmental Biology & Cancer Dept, UCL/GOS Institute of Child Health, London, UK
4. Department of Paediatric Surgery, Great Ormond Street Hospital, London, UK
5. Centre for Outcomes and Experience Research in Children's Health, Illness and Disability (ORCHID), Great Ormond Street Hospital, London, UK

Word count: 2499

Confidential: For Review Only

ABSTRACT

Purpose: The COVID-19 pandemic has resulted in a global health crisis of unparalleled magnitude. The direct risk to the health of children is low. However, disease containment measures have society-wide impacts. This study explored the pandemic experiences of parents of children with oesophageal atresia/tracheo-oesophageal fistula in the UK.

Design: An online forum was conducted using a private group on Facebook, in collaboration with a patient support group. Thematic analysis was used to identify key themes.

Results: The online forum ran between 7th November-18th December 2020 with 109 participants. Themes related to healthcare and non-healthcare impacts. Parents experienced changes and limitations to healthcare access, anxiety regarding health risks, "collateral" damage to well-being because of isolation and an impact on finances and employment. Parents described a transition from worry about direct health risks to concern about the impact of isolation on socialisation and development. A process of risk-benefit analysis led some to transition to a more "normal life", while others continued to isolate. Benefits to their child's health from isolation, positive experiences with remote healthcare and a gradual easing of anxiety were also identified.

Implications and relevance: This study highlights the wide-ranging impact of the COVID-19 pandemic on children and their families. Although focussed on oesophageal atresia/tracheo-oesophageal fistula, the emerging themes will be relevant to many children with complex, chronic health conditions. There are implications for healthcare delivery, including telehealth, during and after the pandemic period. Accurate and consistent messaging is required. Third sector organisations are ideally positioned support this.

Confidential: For Review Only

Confidential: For Review Only

INTRODUCTION

The emergence of SARS-CoV-2 brought about the largest global health crisis for a generation. It is now understood that the direct risk to children from the virus is low, particularly those under 11 years of age.[1 2] Early data indicated that "high risk" groups for severe disease existed[3] and as a result individuals, including children, deemed "extremely clinically vulnerable" were advised to "shield", avoiding contact with others.

One group of vulnerable children are those born with oesophageal atresia/tracheo-oesophageal fistula (OA/TOF). A rare, congenital abnormality, OA/TOF occurs in approximately 1/3500 live births in the UK. While survival rates following surgical repair are excellent, many children experience long-term health challenges.[4] Swallow dysfunction results in feeding difficulties,[5 6] oesophageal stricture is common, particularly in the first year of life, and recurrent respiratory infections and chronic cough are evident in 40-52% of adults and children.[5 7] Hospital readmission with respiratory or gastroenterological issues in the pre-school years is common.[8]

Despite vulnerability to respiratory infection, children with OA/TOF have not experienced severe COVID-19 symptoms.[9] However, disease containment continues to involve restrictions to social contact, education and non-essential business and impacts society beyond the immediate risk to health.[10] Our aim was to understand the wider impact of COVID-19 on children with OA/TOF and their families, learn from their experiences and make recommendations for optimising care for this, and other, rare diseases.

METHOD

A phenomenological approach underpinned use of an online forum to explore parental experiences of accessing healthcare, education and work, managing health risks and social isolation in the UK[11] during the COVID-19 pandemic.

Data were collected using a previously described online forum method.[12] In collaboration with TOFS, the UK support group for OA/TOF, a research-specific, private Facebook group was launched. An experienced member of the TOFS Facebook group, independent of the research team, moderated the forum. This online forum was part of a larger study that was granted ethical approval (20/LO/0098).

Patient and Public Involvement

Patient and public involvement (PPI), through collaboration with TOFS and use of a PPI steering group (including 4 parents) has been integral to study design, recruitment, data analysis and dissemination. Details are provided throughout the methods section.

Participants

Convenience sampling was used to recruit parents of children aged 0-18 years with OA/TOF living in the UK. The TOFS support group advertised participation to their members by email and on their Facebook group. Interested parents were asked to apply to join the research Facebook group, with access granted by the moderator after participants agreed to the group "rules"; acknowledging responses would be anonymised and passed to the research team. Participants provided demographic data via a Survey Monkey questionnaire. These data were used to describe group characteristics but were not linked to individual responses.

Data collection

Questions for the online forum were co-developed with the PPI group and checked by TOFS, ensuring pertinent issues were explored in a sensitive manner and posted individually by the moderator. A new question was posted once there were no further comments to the existing question. The moderator answered participant questions and prompted for clarification or explanation if required. Participants were also able to respond privately to the moderator. To diversify participation, parents not using Facebook, or not wishing to share information on the forum, could participate via email.

Data analysis

All responses were anonymised by the moderator. Thematic analysis was conducted.[13] Data were independently coded by 3 members of the research team, followed by group discussion to agree themes. Two thematic maps were generated. Tables of codes with supporting quotes and the maps were reviewed by the PPI group and 2 other members of the research team providing data validation from different professional and personal perspectives.

RESULTS

The online forum ran from 7/11-18/12/2020. There were 109 members, of whom 65 completed the demographic survey (table 1) and responded to at least one question. An additional six participants responded by email.

Table 1. Demographic data

Relationship to the child	Mother	58 (89%)
	Father	3 (5%)
	Adult with OA/TOF	1 (2%)
	Did not respond	3 (5%)
Ethnicity	White	61 (94%)
	Asian or British Asian	1 (2%)
	Did not respond	3 (5%)
Geographical location	England	55 (82%)
	Scotland	10 (15%)
	Wales	2 (3%)
Age of the child	Under 2 years of age	25 (38%)
	2-4 years of age	33 (34%)
	5-11 years of age	12 (18%)
	Over 12 years of age	3 (5%)
Type of OA/TOF	OA and TOF repaired within a week of birth	55 (85%)
	OA and TOF repaired more than a week after birth	5 (7%)
	OA only	3 (5%)
	TOF only	2 (3%)

Experiences of accessing healthcare

Participants' experiences of accessing healthcare are summarised in Figure 1. Further illustrative quotes are shown in Table 2. Themes were grouped into remote healthcare, delays and cancellations and hospital care.

Table 2. Further illustrative quotes relating to healthcare themes.

https://mc.manuscriptcentral.com/bmjpo

Confidential: For Review Only

Remote healthcare	Benefits of telehealth	They switched it to a telephone appointment. I was really impressed! The consultant was clearly liking the format too, because he suggested the same next year! Oh, and we were 'seen' early - no hanging around for an hour in Clinic 6. It was actually a much better experience than normal.
	Good communication and care	We feel we've had good care and lots of support and all our questions answered as we could write them in an email.
		Still a good level of care and compassion even though over the phone
	Limitations resulting in uncertainty	There appears to have been a real failure to assess the risk of face to face appointments vs the risk to patients of not being seen… had we had a face to face assessment earlier, we might have avoided the blue spells in April/May
		I am concerned that her health has deteriorated slightly over the last month of two and I really would have liked next week's TOF clinic to be face to face as I think the respiratory consultant needs to listen to her chest.
	Organisational difficulties	the consultant phoned a couple of weeks before the expected appointment. This meant we weren't prepared so didn't ask all the questions/mention things we would have wanted to.
		It's been very difficult to get advice about healh issues while the pandemic has been happening it's as if my childs care completely stopped at one point.
Delays and cancellations	Slower progress	We were very concerned about delays to her treatment, and the placing on hold of treatment and check ups, and that the longer term welfare of …. children in particular, was being jeopardised.
		not having face to face it seems a very very slow process to get him weaned off the tube and start him on solids with the help of SaLT
	Escalation to emergency	We had to wait months for an elective scope and dilatation, this became an emergency procedure as was not carried out on time and symptoms persisted.
	Feeling abandoned	The only thing I think has been compromised is support from Speech & Language. They seem to have forgotten us & haven't been very helpful
		On many occasions, I would have taken my child in to see the health visitors had they been open
Inpatient care	Caring alone	…the one parent for hospital stays is incredibly hard. To expect parents not to be with babies when they go in for surgery is really harsh.

Confidential: For Review Only

		When you have a child to care for, then the "one parent" rule means you're trying to do multiple jobs at the same time, which is not efficient for the medics, and not good for ensuring your child gets the care needed.
	Hospital avoidance	…advised to be very cautious and during first lockdown we were told by his consultant she didn't want him anywhere near a hospital (for procedures) as too high risk
	Feeling safe with infection control measures	We had a heart scan at (hospital name) and that was also mid first lockdown and also very safe and clean. Have no complaints at all!

Confidential: For Review Only

Remote healthcare

Access to healthcare changed, with a shift to telehealth (telephone or video) appointments reported by most participants.

Many participants highlighted the ease and convenience of telehealth; no waiting times or travel, not needing to take time off work and not attending busy waiting rooms were benefits. Some parents experienced improved communication with healthcare professionals, having telephone or email contact that was not previously available. Where parents felt connected, remote healthcare was positively received. The value of specialist nurses in achieving good communication was evident.

"we've had huge support from our CNS team who has been amazing throughout the entire journey"

However, limitations of telehealth were also described. Some parents raised concerns that their child's health or development was being compromised. Parents reported feeling disconnected from their healthcare team, due to communication or organisational challenges and the limitations of telehealth appointments. A few expressed concern at how their child would cope with face-to-face appointments after a period of not attending in person.

"I'm worried that my daughter will become withdrawn, nervous, anxious for future appointments"

Delays and cancellations

Delays and cancellations to inpatient and outpatient care were widely reported. Most cancelled appointments had been rebooked.

Participants described concern at anticipated and realised difficulty accessing timely and appropriate care, with stark accounts of feeling abandoned by healthcare services. This was most evident for community services, with Speech and Language Therapy services most frequently cited.

Parents felt that delays impacted directly on their child's health, including escalation of care to an emergency, and impacts on development and growth.

Hospital care

Worries about infection risk and strategies to avoid hospital admission were voiced but all accounts of hospital treatment were positive. Parents felt safe and confident with infection control measures.

The one-parent policy caused the greatest challenge. Participants expressed distress at making decisions regarding surgery and care alone, the absent parent, often the father, being omitted from care, and the challenge of processing information while simultaneously caring for the child.

One parent highlighted the impact of mask-wearing on bonding. Practical challenges, such as not having a parent kitchen and car-parking were also reported. Overall, access to hospital care was reported more positively than community care.

Wider impact of the pandemic

Themes relating to the wider impact of the pandemic are outlined in Figure 2, with further illustrative quotes provided in Table 3.

Table 3. Further illustrative quotes relating to non-healthcare themes

Fear of risk of the child	In the beginning we were extremely concerned and worried about our son catching the virus as months before we had been In hospital for just a cold.
	At the beginning, I think like most people, seeing people on ventilators with respititory issues was extra concerning for TOFs.
Cutting all contact with others	It was extremely stressful, we completely cut off contact with friends and family and shielded, which was difficult and upsetting.
	We completely shielded too to be safe so none of us at home left the house (apart from me walking the dog) March to august
Transition to worry about socialisation/development	Now my main worry is him getting the care and support he needs to develop during the crisis.
Risk vs benefit	My son is 3 and I personally think he needs to social interaction with other kids and family members. It's a risk- but keeping him cooped up is not natural
Feeling reassured	I was relieved when his respiratory consultant explained he no longer needed to shield
	TOFS was ever a great source of support and information by direct posts of latest information and from other parents and their experiences. My son's two consultants were very helpful in putting my mind at rest too.
Moving out of isolation	Now we are back to normal and he is going to nursery we are as careful as possible in terms of hygiene but are living as normal now
	It was a worry with our little boy starting Reception… It did ease our minds a little to know the school was doing everything they could.
Ongoing fear and isolation	I'm not sure if he should be in school or not but I'm keeping him home.
	We weren't advised to shield but did anyway until August and remain cautious. Our TOF is 10 months now - his grandparents have only held him twice and he has yet to meet Uncles and cousins.
Parental mental health impact	I had finally (after poor care at the outset meant we were never seen by the counsellor) started getting help for PTSD.

Confidential: For Review Only

	For the last six weeks I have been signed off because the pressure overwhelmed me. I have been signed off for a further month and referred for counseling by HR.
Impact on work and work-life balance	My husband's ability to work was affected since he was at home with a screaming child usually with him (I was entertaining the 2 year old).
	More work, less time, more pressure to do housework and make the meals due to being at home - and having to create time to make sure we were getting lots of exercise- all his health needs are managed by me.

Fear of risk to the child and cutting contact

Fears for their child's health were expressed almost universally. Several parents described an overwhelming fear that their child was going to die.

"I couldn't shift the feeling again that we that we were going to lose him"

This led to most participants cutting all contact with others outside their household; some following UK Government advice to "shield", others without this advice. Whether and when children were advised to shield varied. Some received instruction at the start of the pandemic, some after a couple of months and some not at all. Where shielding advice was not immediate many participants sought information from healthcare teams and the TOFS support group. Participants identified the TOFS website and online peer support as particularly helpful.

"The TOFS Facebook group is the only place where I have seen useful information about our TOFS"

Shielding experiences were varied. Some felt gratitude for the family time. For others, balancing work with children at home or the social isolation resulted in high levels of stress.

Transitioning to new worries

Many parents transitioned from worrying about their child's health to worrying about socialisation and development. They described balancing the health benefits of isolation with the risks to well-being.

Participants highlighted the challenge of assessing the risks/benefits of school or childcare attendance. Good communication with the school/nursery and trust in the infection control procedures facilitated attendance. Some reported that infection control measures prevented in-person staff training, disrupting transition to school/nursery. One child was unable to access longer hours in nursery as the parent was unable to go on site to gastrostomy-feed her child. Two parents highlighted to school staff their child's chronic OA/TOF-related cough to differentiate it from an infectious, COVID-19 related cough.

"…wanted to explain that her TOF cough was normal for her…It wasn't ideal having to try to explain at a distance at the door, but I didn't want her new teacher to be alarmed (or other kids in the class) when she coughed."

Moving out of isolation and ongoing fear and isolation

Fear led to complete isolation for almost all participants initially, but as the pandemic progressed experiences diverged. For some, increased knowledge provided reassurance. This, coupled with increasing concern about the social and emotional effects of isolation, prompted transition to a more

Confidential: For Review Only

"normal life", albeit within society-wide restrictions. For others, increased information and knowledge was not reassuring, resulting in continued isolation and anxiety, including continued home-learning.

Health and development benefits

Strikingly, many parents reported benefits of social isolation, highlighting reduced illness and hospitalisations, improved weight gain and improvements to general development.

Impact on parental mental health and work-life balance

Parents made direct reference to the impact on their own mental health, with a small number requiring professional support for anxiety or post-traumatic stress disorder.

Combining childcare with home working, managing with limited space and an increased burden of domestic tasks were stress-inducing. Financial hardship was reported. The ability to work was affected by the need to look after children, avoidance of social contact to keep their child safe and parental mental health difficulties.

DISCUSSION

The online forum allowed for timely gathering of parental insights into the impact of the COVID-19 pandemic on children with OA/TOF. The method engaged a large number of parents from a rare disease cohort, avoided face-to-face contact and minimised burden by allowing for asynchronous participation.

Access to healthcare during the pandemic has been shaped by infection prevention and control measures, limiting face-to-face contact, and prioritisation of "essential" services. This study highlights the significant impact these changes have had.

Telehealth was widely and rapidly adopted. No parents reported having accessed telehealth prior to the pandemic. Benefits, including access to specialist services from geographically distant locations and reduced costs and time to patients reported in this study, have long been recognised.[14] Recent technological advances in mobile communication, software and high-speed internet have increased feasibility of telehealth[14] and effective use in paediatric surgical conditions has been demonstrated.[15 16]

While many parents reported receiving good care remotely, wanting this to continue post-pandemic, some felt disconnected from their healthcare team and that care was suboptimal. These views are echoed by clinicians.[17] Our study design prevented linking demographic data to individual responses. However, diagnostic complexity and the age of the child varied within the group. Younger children with OA/TOF tend to have more challenging health needs with post-operative morbidity is greater for some.[8 18] We propose satisfaction with telehealth was greater for those at a stable point in their care than those with specific concerns. Dissatisfaction was not reported in other, pre-pandemic studies evaluating use of telehealth with paediatric surgical patients.[15 19] This may be due to the speed with which telehealth was rolled out, with blanket, rather than targeted, use. It is likely that use of telehealth will continue post-pandemic. The needs of the individual and family must be considered to ensure appropriate use. Providers must address the training needs of the current and future workforce to optimise remote examination and develop effective communication.

Prioritisation of care and redeployment of staff seemingly impacted most on community services. Access to community Speech and Language Therapy appeared particularly poor. The Royal College of Speech and Language Therapists has highlighted the negative impact of redeployment on waiting lists

https://mc.manuscriptcentral.com/bmjpo

Confidential: For Review Only

and access to care.[20] Recent increases in COVID-19 related hospital admissions, the impact of "long-COVID" and staff sickness all continue to threaten access to services. This study highlights the impact that a lack of community services has on children's physical health and development but also on parental mental health. Innovative solutions are required to protect non-acute services in periods of extreme resource demand to minimise "collateral" damage.

The wider harms caused by society wide "lockdown" have been well summarised[10] and are reflected in our findings; fear and anxiety, displaced non-COVID care, social isolation, stress and loss of income were all reported. Similar themes emerged from research exploring the experiences of parents caring for children with cancer.[21] Our findings highlight the diversity of experiences, typified by divergent management of anxiety that enabled some to transition to a more normal life, while others continued to isolate. Although individual differences in risk evaluation are inevitable, clear communication of the best available evidence would mitigate unnecessary isolation.

Parents highlighted the challenge of obtaining information about risks to their child. Not all children were identified as "extremely clinically vulnerable" and advice to shield was mixed. Mixed messages and the need for parents to make their own decisions increased anxiety, also identified in parents of children with cancer.[21] Support groups play an important role in provision of disease-specific information. While individualised assessment may be required, communication between the Government and/or healthcare providers and the charitable sector should be leveraged as a means of sharing accurate and consistent information.

Many parents described the positive impact that isolation had on their child's health and growth. Exposure to common viruses and other infections is usually an inescapable part of childhood and supports development of a well-functioning immune system[22] but can necessitate hospital treatment for vulnerable children. The long-term impact of not being exposed is unknown. However, clinicians should be aware of the potential challenge that some parents will face in supporting normal childhood activity with the knowledge that avoidance may improve health post-pandemic.

Chronic cough is common in children with OA/TOF.[7 23] Interestingly, difficulty differentiating infective coughing from the child's usual cough was not commonly reported and was not hindering school attendance. TOFS provide excellent resources for families to educate about chronic "TOF cough", limiting misunderstanding and empowering parents to advocate appropriately.

Limitations

Despite efforts to facilitate wider involvement, participants were overwhelmingly female, white parents of pre-school children, likely reflecting those most commonly using Facebook and engaging with a support group. We acknowledge that although a wide range of experiences were described, they may not be reflective of the whole OA/TOF community.

Description of group rather than individual demographics supported anonymity but prevented sub-analysis by OA/TOF type or age. Future research should identify whether such factors impact on satisfaction with telehealth and the assessment of risk.

Conclusion

This study demonstrates the indirect impact that COVID-19 has had on children with a chronic health condition. Parents experienced changes and limitations to healthcare access, anxiety regarding health risks, "collateral" damage to well-being because of isolation and an impact on finances and employment. Benefits to their child's health from isolation, positive experiences with remote healthcare and a gradual easing of anxiety were also identified.

https://mc.manuscriptcentral.com/bmjpo

Confidential: For Review Only

Confidential: For Review Only

What is already known on this topic

- Direct health risk to children from COVID-19 is low but there is a high risk of "collateral" damage from strategies required to contain the virus.
- Patient support groups can be powerful allies in providing accurate and consistent messages, that is particularly useful to those with rare diseases.
- Social media can facilitate rapid and effective data collection in a rare disease cohort.

What this study adds

- Pandemic-related reduction in healthcare provision has impacted disproportionately on the health and development of children with chronic conditions and on parental mental health.
- Urgent assessment of telehealth policies are required to effectively embed this change to service provision in paediatric practice and ensure appropriate use.
- Isolation has resulted in exceptionally low exposure to usual childhood infections resulting in improved health for children vulnerable to respiratory infection.

Confidential: For Review Only

Acknowledgments

The authors wish to thank Joanne Winspear for moderating the Facebook group. Thank you to Diane Stephens and the team at TOFS for their support and guidance in developing the research and facilitating recruitment. Thank you to Neil Carding, Susan Morris, Hayley Ramm, Julia Faulkner, Kelly Lamour and Helen Marks for their invaluable contributions in the PPI steering group. Finally, thank you to all the parents who gave their time to share their experiences.

Funding

Alexandra Stewart is currently undertaking a PhD funded by the National Institute for Health Research (NIHR) (award reference: ICA-CDRF-2018-04-ST2-042).

This publication indicates independent research funded by the National Institute for Health Research (NIHR). The views expressed are those of the authors and not necessarily those of the NHS, the NIHR or the Department of Health and Social Care.

Research at Great Ormond Street Hospital for Children NHS Trust is supported by the NIHR GOS/ICH Biomedical Research Centre.

Confidential: For Review Only

References

1. Ladhani SN, Amin-Chowdhury Z, Davies HG, et al. COVID-19 in children: analysis of the first pandemic peak in England. *Arch Dis Child* 2020;105(12):1180-85. doi: 10.1136/archdischild-2020-320042 [published Online First: 2020/08/17]
2. Control. ECfDPa. COVID-19 in children and the role of school settings in transmission - first update. Stockholm, 2020.
3. Yang J, Zheng Y, Gou X, et al. Prevalence of comorbidities and its effects in patients infected with SARS-CoV-2: a systematic review and meta-analysis. *Int J Infect Dis* 2020;94:91-95. doi: 10.1016/j.ijid.2020.03.017 [published Online First: 2020/03/17]
4. van Lennep M, Singendonk MMJ, Dall'Oglio L, et al. Oesophageal atresia. *Nat Rev Dis Primers* 2019;5(1):26. doi: 10.1038/s41572-019-0077-0 [published Online First: 2019/04/20]
5. Connor MJ, Springford LR, Kapetanakis VV, et al. Esophageal atresia and transitional care--step 1: a systematic review and meta-analysis of the literature to define the prevalence of chronic long-term problems. *Am J Surg* 2015;209(4):747-59. doi: 10.1016/j.amjsurg.2014.09.019 [published Online First: 2015/01/22]
6. Mahoney L, Rosen R. Feeding Difficulties in Children with Esophageal Atresia. *Paediatr Respir Rev* 2016;19:21-7. doi: 10.1016/j.prrv.2015.06.002 [published Online First: 2015/07/15]
7. Kovesi T. Aspiration Risk and Respiratory Complications in Patients with Esophageal Atresia. *Front Pediatr* 2017;5:62. doi: 10.3389/fped.2017.00062 [published Online First: 2017/04/20]
8. Scheider A, Blanc, S., Bonnard, A et al. Results from the French national esophageal atresia register: 1 yr outcome. *Orphanet journal of rare diseases* 2014;9:206.
9. TOFS. 2021 [Available from: https://www.tofs.org.uk/news/2020/03/coronavirus-update.aspx accessed 28th January 2021.
10. Douglas M, Katikireddi SV, Taulbut M, et al. Mitigating the wider health effects of covid-19 pandemic response. *BMJ* 2020;369:m1557. doi: 10.1136/bmj.m1557 [published Online First: 2020/04/29]
11. Neubauer BE, Witkop CT, Varpio L. How phenomenology can help us learn from the experiences of others. *Perspect Med Educ* 2019;8(2):90-97. doi: 10.1007/s40037-019-0509-2 [published Online First: 2019/04/07]
12. Wray J, Brown K, Tregay J, et al. Parents' Experiences of Caring for Their Child at the Time of Discharge After Cardiac Surgery and During the Postdischarge Period: Qualitative Study Using an Online Forum. *Journal of Medical Internet Research* 2018;20(5) doi: 10.2196/jmir.9104
13. Braun V, Clarke V. Using thematic analysis in psychology. *Qualitative research in psychology* 2006;3(2):77-101.
14. Dorsey ER, Topol EJ. State of Telehealth. *N Engl J Med* 2016;375(2):154-61. doi: 10.1056/NEJMra1601705 [published Online First: 2016/07/15]
15. Dean P, O'Donnell M, Zhou L, et al. Improving value and access to specialty medical care for families: a pediatric surgery telehealth program. *Can J Surg* 2019;62(6):436-41. doi: 10.1503/cjs.005918 [published Online First: 2019/11/30]
16. Goedeke J, Ertl A, Zoller D, et al. Telemedicine for pediatric surgical outpatient follow-up: A prospective, randomized single-center trial. *J Pediatr Surg* 2019;54(1):200-07. doi: 10.1016/j.jpedsurg.2018.10.014 [published Online First: 2018/10/23]
17. Kemp MT, Liesman DR, Williams AM, et al. Surgery Provider Perceptions on Telehealth Visits During the COVID-19 Pandemic: Room for Improvement. *J Surg Res* 2020;260:300-06. doi: 10.1016/j.jss.2020.11.034 [published Online First: 2020/12/29]
18. Svoboda E, Fruithof J, Widenmann-Grolig A, et al. A patient led, international study of long term outcomes of esophageal atresia: EAT 1. *Journal of Pediatric Surgery* 2018;53(4):610-15.
19. Kohler JE, Falcone RA, Jr., Fallat ME. Rural health, telemedicine and access for pediatric surgery. *Curr Opin Pediatr* 2019;31(3):391-98. doi: 10.1097/MOP.0000000000000763 [published Online First: 2019/05/16]

Confidential: For Review Only

20. Therapists RCoSaL. Redeployment of staff and impact on outcomes for service. London, 2020:1-5.
21. Darlington AE, Morgan JE, Wagland R, et al. COVID-19 and children with cancer: Parents' experiences, anxieties and support needs. *Pediatr Blood Cancer* 2021;68(2):e28790. doi: 10.1002/pbc.28790 [published Online First: 2020/11/22]
22. Ball TM, Castro-Rodriguez JA, Griffith KA, et al. Siblings, day-care attendance, and the risk of asthma and wheezing during childhood. *New England journal of medicine* 2000;343(8):538-43.
23. Sadreameli SC, McGrath-Morrow SA. Respiratory Care of Infants and Children with Congenital Tracheo-Oesophageal Fistula and Oesophageal Atresia. *Paediatr Respir Rev* 2016;17:16-23. doi: 10.1016/j.prrv.2015.02.005 [published Online First: 2015/03/25]

Confidential: For Review Only

Figure 1. Thematic map: healthcare impact

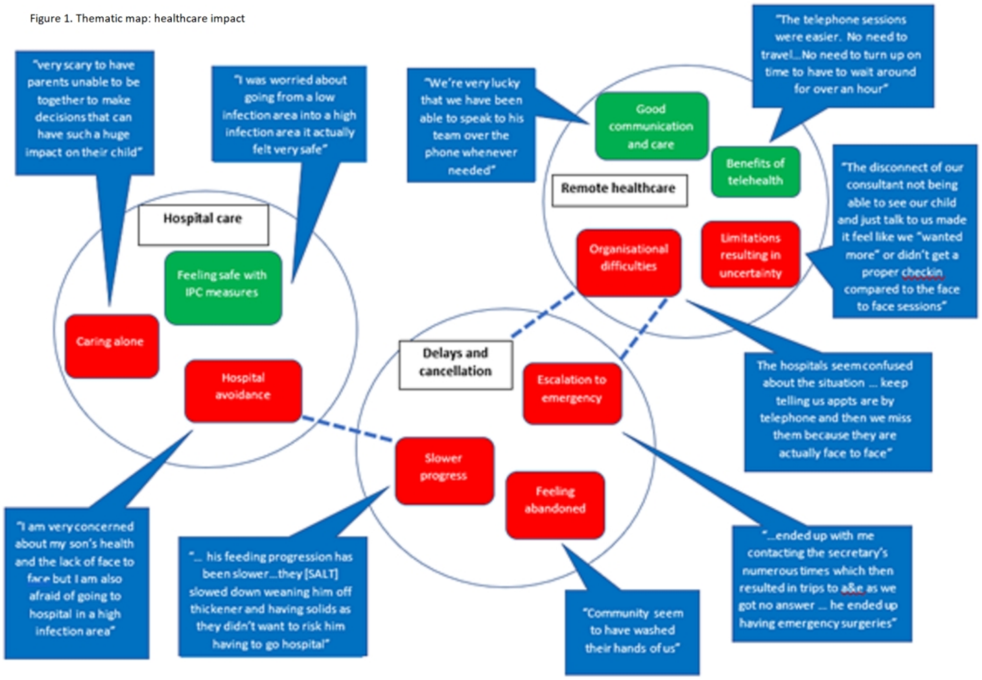

Legend: Green boxes = facilitative factors, Red boxes = prohibitative factors

Figure 2. Thematic map: non-healthcare impact

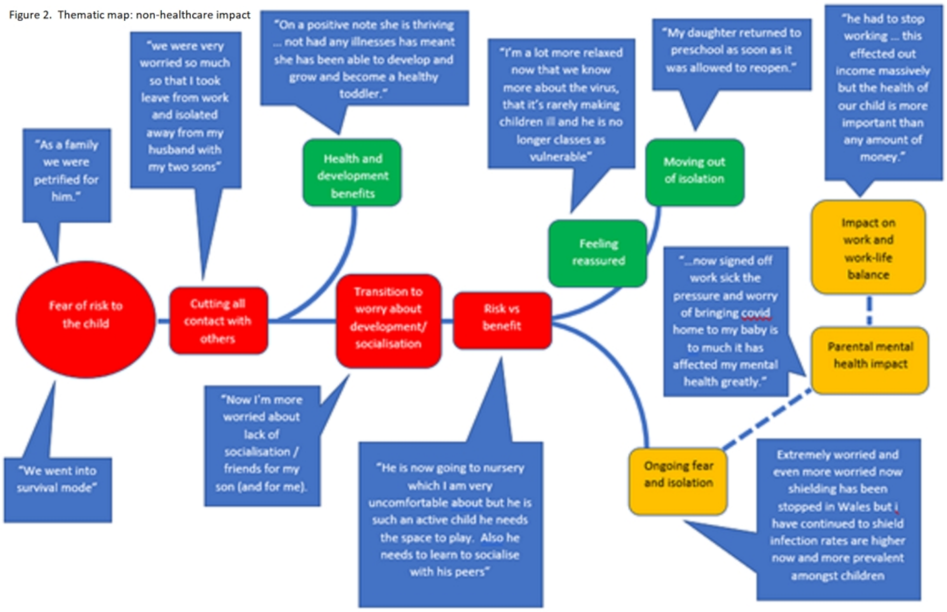

BMJ Paediatrics Open

The COVID-19 pandemic experiences of parents caring for children with oesophageal atresia/tracheo-oesophageal fistula

Journal:	*BMJ Paediatrics Open*
Manuscript ID	bmjpo-2021-001077.R1
Article Type:	Original research
Date Submitted by the Author:	27-Apr-2021

Complete List of Authors:	Stewart, Alexandra; Great Ormond Street Hospital For Children NHS Trust, Department of Speech and Language Therapy; University College London, Department of Language and Cognition<br>Smith, Christina; University College London, Language and Cognition<br>Eaton, Simon; University College London Institute of Child Health, Paediatric Surgery<br>De Coppi, Paolo; Great Ormond Street Hospital For Children NHS Trust, General Surgery; University College London Institute of Child Health, Developmental Biology and Cancer<br>Wray, Jo; Great Ormond Street Hospital For Children NHS Trust, Centre for Outcomes and Experience Research in Children's Health, Illness and Disability (ORCHID)

Keywords:	COVID-19, Qualitative research

SCHOLARONE™
Manuscripts

I, the Submitting Author has the right to grant and does grant on behalf of all authors of the Work (as defined in the below author licence), an exclusive licence and/or a non-exclusive licence for contributions from authors who are: i) UK Crown employees; ii) where BMJ has agreed a CC-BY licence shall apply, and/or iii) in accordance with the terms applicable for US Federal Government officers or employees acting as part of their official duties; on a worldwide, perpetual, irrevocable, royalty-free basis to BMJ Publishing Group Ltd ("BMJ") its licensees and where the relevant Journal is co-owned by BMJ to the co-owners of the Journal, to publish the Work in this journal and any other BMJ products and to exploit all rights, as set out in our licence.

The Submitting Author accepts and understands that any supply made under these terms is made by BMJ to the Submitting Author unless you are acting as an employee on behalf of your employer or a postgraduate student of an affiliated institution which is paying any applicable article publishing charge ("APC") for Open Access articles. Where the Submitting Author wishes to make the Work available on an Open Access basis (and intends to pay the relevant APC), the terms of reuse of such Open Access shall be governed by a Creative Commons licence – details of these licences and which Creative Commons licence will apply to this Work are set out in our licence referred to above.

Other than as permitted in any relevant BMJ Author's Self Archiving Policies, I confirm this Work has not been accepted for publication elsewhere, is not being considered for publication elsewhere and does not duplicate material already published. I confirm all authors consent to publication of this Work and authorise the granting of this licence.

Confidential: For Review Only

The COVID-19 pandemic experiences of parents caring for children with oesophageal atresia/tracheo-oesophageal fistula

Stewart, A.[1,2], Smith, C.[2], Eaton, S.[3], De Coppi, P.[3,4] and Wray, J. [5].

Corresponding author: Alexandra Stewart, Department of Speech and Language Therapy, Great Ormond Street Hospital, Great Ormond Street, London, WC1N 3JH.

Email: Alex.stewart@gosh.nhs.uk

1. Department of Speech and Language Therapy, Great Ormond Street Hospital, London, UK
2. Department of Language and Cognition, Division of Psychology and Language Sciences, University College London, London, UK
3. Developmental Biology & Cancer Dept, UCL/GOS Institute of Child Health, London, UK
4. Department of Paediatric Surgery, Great Ormond Street Hospital, London, UK
5. Centre for Outcomes and Experience Research in Children's Health, Illness and Disability (ORCHID), Great Ormond Street Hospital, London, UK

Word count: 2803

Confidential: For Review Only

ABSTRACT

Purpose: The COVID-19 pandemic has resulted in a global health crisis of unparalleled magnitude. The direct risk to the health of children is low. However, disease containment measures have society-wide impacts. This study explored the pandemic experiences of parents of children with oesophageal atresia/tracheo-oesophageal fistula in the UK.

Design: A phenomenological approach underpinned use of an asynchronous online forum method, in collaboration with a patient support group. Data were evaluated using thematic analysis.

Results: The online forum ran between 7th November-18th December 2020 with 109 participants.

Pandemic experiences were divided into themes relating to healthcare and disease containment. Participants described positive experiences with remote healthcare but identified limitations. Delays and cancellations led to escalation of care to an emergency level, slower developmental progress, and feelings of being abandoned by services. Inpatient care was perceived as safe but caring alone was emotionally and practically challenging. Disease containment themes revealed anxiety regarding health risks, "collateral" damage to well-being because of isolation and an impact on finances and employment. Parents described a transition from worry about direct health risks to concern about the impact of isolation on socialisation and development. A process of risk-benefit analysis led some to transition to a more "normal life", while others continued to isolate. Benefits to their child's health from isolation were reported.

Conclusions: Parents experiences of caring for a child with OA/TOF during the pandemic were varied. Rapid adoption of telehealth has demonstrated the enormous potential of remote healthcare delivery but requires refinement to meet the needs of the individual. Future pandemic planning should aim to retain community health care services to avoid escalation of care to an emergency, manage chronic and developmental concerns and support parental well-being. Accurate and consistent, disease-specific information is highly valued by parents. Third sector organisations are ideally positioned to support this.

Confidential: For Review Only

INTRODUCTION

The emergence of SARS-CoV-2 brought about the largest global health crisis for a generation

Although it is now suggested that children are approximately 50% less likely to be infected than adults, account for 1-5% of cases worldwide [1 2] and rarely experience severe disease [3], early data indicated that "high risk" groups for severe disease existed.[4] As a result children deemed "extremely clinically vulnerable" were advised to "shield", avoiding all contact with others to minimise their risk of being infected.

One group of vulnerable children are those born with oesophageal atresia/tracheo-oesophageal fistula (OA/TOF). A rare, congenital abnormality, OA/TOF occurs in approximately 1/3500 live births in the UK that results in a blind ending oesophageal pouch and/or an anomalous connection between the trachea and oesophagus. While survival rates following surgical repair are excellent, many children experience long-term health challenges:[5] swallow dysfunction and feeding difficulties in approximately 80%,[6 7] gastro-oesophageal reflux and oesophagitis in up to 70%, [5] recurrent respiratory infections and chronic cough in 40-52%.[6 8] Approximately 50% of children have other congenital abnormalities, most commonly cardiac abnormalities. [5] Hospital readmission with respiratory or gastroenterological issues in the pre-school years is common.[9]

Despite vulnerability to respiratory infection, children with OA/TOF have not experienced severe COVID-19 symptoms.[10] However, disease containment continues to involve restrictions to social contact, education and non-essential business and impacts society beyond the immediate risk to health.[11] Our aim was to describe parental lived experiences of caring for a child with OA/TOF during the COVID-19 pandemic with the following specific objectives:

1. To describe experiences of accessing healthcare and medical advice
2. To describe parental experiences of disease containment measures and their impact
3. To learn from their experiences and make recommendations for delivery of care for this rare disease.

METHOD

A phenomenological approach underpinned use of an online forum to explore parental experiences of accessing healthcare and the impact of disease containment measures in the UK during the COVID-19 pandemic.[12]

Data were collected using a previously described online forum method and detailed in the supplementary material.[13]

In collaboration with TOFS, the UK support group for OA/TOF, a research-specific, private Facebook group was launched. An experienced member of the TOFS Facebook group, independent of the research team, moderated the forum. This online forum was part of a larger study that was granted ethical approval (20/LO/0098).

Patient and Public Involvement

Patient and public involvement (PPI), through collaboration with TOFS and use of a PPI steering group (including 4 parents) has been integral to study design, recruitment, data analysis and dissemination. Details are provided throughout the methods section.

Confidential: For Review Only

Participants

Convenience sampling was used to recruit parents of children aged 0-18 years with OA/TOF living in the UK. The TOFS support group advertised participation to their members by email and on their Facebook group. Interested parents were asked to apply to join the research Facebook group, with access granted by the moderator after participants consented to participation by agreeing the group "rules"; acknowledging responses would be anonymised and passed to the research team. Participants provided demographic data via a link to a separate Survey Monkey questionnaire. These data were used to describe group characteristics but were not linked to individual responses.

Data collection

Questions for the online forum, provided in table 1, were co-developed with the PPI group and checked by TOFS, ensuring pertinent issues were explored in a sensitive manner. and posted Questions were posted individually by the moderator and participants responded by posting a "comment". Data saturation was assumed once no further comments were being made. A new question was then posted. The moderator answered participant questions, prompted for clarification, and invited further responses if required. Participants were able to respond to other's comments. Participants were also able to respond privately to the moderator rather than posting to the whole group. To diversify participation, parents not using Facebook, or not wishing to share information on the forum, could participate via email.

Table 1. Online forum questions

1. If you were expecting any healthcare appointments during the COVID-19 pandemic, what changes or disruptions have you experienced to your/your child's normal hospital or community care?<br>**Follow up questions**<br>• Are you concerned about the impact of any changes on your health/the health of your child/the person you care for?<br>• What did the services do well under the circumstances?<br>• What did not work well?
2. Thinking back to earlier this year. What were you worried about at the beginning of the pandemic? Has this changed over the last 6 months?
3. Were you advised to shield? If so, do you feel that this has had an impact on your child or family?
4. What was your experience of seeking support or advice during the pandemic for OA/TOF related concerns? Who provided you with the most useful information?
5. How have any OA/TOF related difficulties impacted on your child's transitioned back into nursery/childminder/school/college/university, during the pandemic? Please think about the impact on you as a parent and the impact on your child. How have you managed these?
6. During the first wave of the pandemic did your child's health impact on your ability to work? Are there ongoing challenges relating to your ability to work?

Data analysis

https://mc.manuscriptcentral.com/bmjpo

Confidential: For Review Only

All responses were anonymised by the moderator and sent to the research team as a Word document. Thematic analysis was conducted.[14] Data were independently coded by 3 members of the research team, followed by group discussion to agree themes. Two thematic maps were generated. Tables of codes with supporting quotes and the maps were reviewed by the PPI group and 2 other members of the research team providing data triangulation from different professional and personal perspectives.

RESULTS

The online forum ran from 7/11-18/12/2020. There were 109 members, of whom 65 completed the demographic survey (table 2) and responded to at least one question. An additional six participants responded by email.

Table 2. Demographic data

Relationship to the child	Mother	58 (89%)
	Father	3 (5%)
	Adult with OA/TOF	1 (2%)
	Did not respond	3 (5%)
Ethnicity	White	61 (94%)
	Asian or British Asian	1 (2%)
	Did not respond	3 (5%)
Geographical location	England	55 (82%)
	Scotland	10 (15%)
	Wales	2 (3%)
Age of the child	Under 2 years of age	25 (38%)
	2-4 years of age	33 (34%)
	5-11 years of age	12 (18%)
	Over 12 years of age	3 (5%)
Type of OA/TOF	OA and TOF repaired within a week of birth	55 (85%)
	OA and TOF repaired more than a week after birth	5 (7%)
	OA only	3 (5%)
	TOF only	2 (3%)

Experiences of accessing healthcare

Participants' experiences of accessing healthcare are summarised in Figure 1. Further illustrative quotes are shown in Table 3. Themes were grouped into remote healthcare, delays and cancellations and hospital care.

Table 3. Further illustrative quotes relating to healthcare themes.

Remote healthcare	Benefits of telehealth	They switched it to a telephone appointment. I was really impressed! The consultant was clearly liking the format too, because he suggested the same next year! Oh, and we were 'seen' early - no hanging around for an hour in Clinic 6. It was actually a much better experience than normal.

Confidential: For Review Only

	Good communication and care	We feel we've had good care and lots of support and all our questions answered as we could write them in an email.
		Still a good level of care and compassion even though over the phone
	Limitations resulting in uncertainty	There appears to have been a real failure to assess the risk of face to face appointments vs the risk to patients of not being seen… had we had a face to face assessment earlier, we might have avoided the blue spells in April/May
		I am concerned that her health has deteriorated slightly over the last month of two and I really would have liked next week's TOF clinic to be face to face as I think the respiratory consultant needs to listen to her chest.
	Organisational difficulties	the consultant phoned a couple of weeks before the expected appointment. This meant we weren't prepared so didn't ask all the questions/mention things we would have wanted to.
		It's been very difficult to get advice about healh issues while the pandemic has been happening it's as if my childs care completely stopped at one point.
Delays and cancellations	Slower progress	We were very concerned about delays to her treatment, and the placing on hold of treatment and check ups, and that the longer term welfare of …. children in particular, was being jeopardised.
		not having face to face it seems a very very slow process to get him weaned off the tube and start him on solids with the help of SaLT
	Escalation to emergency	We had to wait months for an elective scope and dilatation, this became an emergency procedure as was not carried out on time and symptoms persisted.
	Feeling abandoned	The only thing I think has been compromised is support from Speech & Language. They seem to have forgotten us & haven't been very helpful
		On many occasions, I would have taken my child in to see the health visitors had they been open
Inpatient care	Caring alone	…the one parent for hospital stays is incredibly hard. To expect parents not to be with babies when they go in for surgery is really harsh.
		When you have a child to care for, then the "one parent" rule means you're trying to do multiple jobs at the same time, which is not efficient for the medics, and not good for ensuring your child gets the care needed.
	Hospital avoidance	…advised to be very cautious and during first lockdown we were told by his consultant she didn't

Confidential: For Review Only

		want him anywhere near a hospital (for procedures) as too high risk
	Feeling safe with infection control measures	We had a heart scan at (hospital name) and that was also mid first lockdown and also very safe and clean. Have no complaints at all!

Remote healthcare

Access to healthcare changed, with a shift to telehealth (telephone or video) appointments reported by most participants.

Benefits to telehealth were identified by many participants: no waiting times or travel, not needing to take time off work and not attending busy waiting rooms. Some parents experienced good, and even improved, communication with healthcare professionals, having telephone or email contact that was not previously available. Where parents felt connected, remote healthcare was positively received. The value of specialist nurses in achieving good communication was evident.

"we've had huge support from our CNS team who has been amazing throughout the entire journey"

However, limitations resulting in uncertainty were also described. Some parents raised concerns that their child's health or development was being compromised. Parents reported feeling disconnected from their healthcare team, due to communication or organisational challenges and the limitations of telehealth appointments. A few expressed concern at how their child would cope with face-to-face appointments after a period of not attending in person.

"I'm worried that my daughter will become withdrawn, nervous, anxious for future appointments"

Delays and cancellations

Delays and cancellations to inpatient and outpatient care were widely reported. Most cancelled appointments had been rebooked.

Participants described concern at anticipated and realised difficulty accessing timely and appropriate care, with stark accounts of feeling abandoned by healthcare services. This was most evident for community services, with Speech and Language Therapy services most frequently cited.

Parents felt that delays impacted directly on their child's health, including escalation of care to an emergency, and slower developmental progress.

Hospital care

Hospital avoidance due to concerns about infection risk were reported but all accounts of hospital treatment were positive. Parents felt safe with infection prevention and control measures.

Caring alone, due to one parent policies, caused the greatest challenge. Participants described distress making decisions regarding care, including surgery alone, the absent parent being omitted from care , and the challenge of processing information while simultaneously looking after the child.

One parent highlighted the impact of mask-wearing on bonding. Practical challenges, such as not having a parent kitchen and car-parking were also reported. Overall, access to hospital care was reported more positively than community care.

Experiences of disease containment and their impacts

Confidential: For Review Only

Themes relating to disease containment are outlined in Figure 2, with further illustrative quotes provided in Table 4.

Table 4. Further illustrative quotes relating to disease containment

Fear of risk of the child	In the beginning we were extremely concerned and worried about our son catching the virus as months before we had been In hospital for just a cold.
	At the beginning, I think like most people, seeing people on ventilators with respiratory issues was extra concerning for TOFs.
Cutting all contact with others	It was extremely stressful, we completely cut off contact with friends and family and shielded, which was difficult and upsetting.
	We completely shielded too to be safe so none of us at home left the house (apart from me walking the dog) March to august
Transition to worry about socialisation/development	Now my main worry is him getting the care and support he needs to develop during the crisis.
Risk vs benefit	My son is 3 and I personally think he needs to social interaction with other kids and family members. It's a risk- but keeping him cooped up is not natural
Feeling reassured	I was relieved when his respiratory consultant explained he no longer needed to shield
	TOFS was ever a great source of support and information by direct posts of latest information and from other parents and their experiences. My son's two consultants were very helpful in putting my mind at rest too.
Moving out of isolation	Now we are back to normal and he is going to nursery we are as careful as possible in terms of hygiene but are living as normal now
	It was a worry with our little boy starting Reception… It did ease our minds a little to know the school was doing everything they could.
Ongoing fear and isolation	I'm not sure if he should be in school or not but I'm keeping him home.
	We weren't advised to shield but did anyway until August and remain cautious. Our TOF is 10 months now - his grandparents have only held him twice and he has yet to meet Uncles and cousins.
Parental mental health impact	I had finally (after poor care at the outset meant we were never seen by the counsellor) started getting help for PTSD.
	For the last six weeks I have been signed off because the pressure overwhelmed me. I have been signed off for a further month and referred for counseling by HR.
Impact on work and work-life balance	My husband's ability to work was affected since he was at home with a screaming child usually with him (I was entertaining the 2 year old).
	More work, less time, more pressure to do housework and make the meals due to being at home - and having to create time to make sure we were getting lots of exercise- all his health needs are managed by me.

Confidential: For Review Only

Fear of risk to the child and cutting contact

Fears for their child's health were expressed almost universally. Several parents described an overwhelming fear that their child was going to die.

"I couldn't shift the feeling again that we that we were going to lose him"

This led to most participants cutting all contact with others outside their household; some following UK Government advice to "shield", others without this advice. Whether and when children were advised to shield varied. Some received instruction at the start of the pandemic, some after a couple of months and some not at all. Where shielding advice was not immediate many participants sought information from healthcare teams and the TOFS support group. Participants identified the TOFS website and online peer support as particularly helpful.

"The TOFS Facebook group is the only place where I have seen useful information about our TOFS"

Shielding experiences were varied. Some felt gratitude for the family time. For others, balancing work with children at home or the social isolation resulted in high levels of stress.

Transitioning to new worries

Many parents transitioned from worrying about their child's health to worrying about socialisation and development. They described balancing the health benefits of isolation with the risks to well-being.

Participants highlighted the burden of assessing the risks/benefits of school or childcare attendance. Good communication with the school/nursery and trust in the infection control procedures facilitated attendance. Some reported that infection control measures prevented in-person staff training, disrupting transition to school/nursery. One child was unable to access longer hours in nursery as the parent was unable to go on site to gastrostomy-feed her child. Two parents highlighted to school staff their child's chronic OA/TOF-related cough to differentiate it from an infectious, COVID-19 related cough.

"…wanted to explain that her TOF cough was normal for her…It wasn't ideal having to try to explain at a distance at the door, but I didn't want her new teacher to be alarmed (or other kids in the class) when she coughed."

Moving out of isolation and ongoing fear and isolation

Fear led to complete isolation for almost all participants initially, but as the pandemic progressed experiences diverged. For some, increased knowledge provided reassurance. This, coupled with increasing concern about the social and emotional effects of isolation, prompted transition to a more "normal life", albeit within society-wide restrictions. For others, increased information and knowledge was not reassuring, resulting in continued isolation and anxiety, including continued home-learning.

Health and development benefits

Strikingly, many parents reported benefits of social isolation, highlighting reduced illness and hospitalisations, improved weight gain and improvements to general development.

Impact on parental mental health and work-life balance

https://mc.manuscriptcentral.com/bmjpo

Parents made direct reference to the impact that isolation as a result of disease containment measures, difficulty accessing healthcare and anxiety about the health risk to their child had on their own mental health. A small number required professional support for anxiety or post-traumatic stress disorder.

Combining childcare with home working, managing with limited space and an increased burden of domestic tasks were stress-inducing. Financial hardship was reported. The ability to work was affected by the need to look after children, avoidance of social contact to keep their child safe and parental mental health difficulties.

DISCUSSION

The online forum allowed for timely gathering of parental insights into the impact of the COVID-19 pandemic on children with OA/TOF. The method engaged a large number of parents from a rare disease cohort, avoided face-to-face contact and minimised burden by allowing for asynchronous participation.

Access to healthcare during the pandemic has been shaped by infection prevention and control measures, limiting face-to-face contact, and prioritisation of "essential" services. This study highlights the significant impact these changes have had.

Telehealth was widely and rapidly adopted. No parents reported having accessed telehealth prior to the pandemic. Benefits, including access to specialist services from geographically distant locations and reduced costs and time to patients reported in this study, have long been recognised.[15] Recent technological advances in mobile communication, software and high-speed internet have increased feasibility of telehealth[15] and effective use in paediatric surgical conditions has been demonstrated.[16 17]

While many parents reported receiving good care remotely, wanting this to continue post-pandemic, some felt disconnected from their healthcare team and that care was suboptimal. These views are echoed by clinicians.[18] Our study design prevented linking demographic data to individual responses. However, diagnostic complexity and the age of the child varied within the group. Younger children with OA/TOF tend to have more challenging health needs with post-operative morbidity is greater for some.[9 19] We propose satisfaction with telehealth was greater for those at a stable point in their care than those with specific concerns. Dissatisfaction was not reported in other, pre-pandemic studies evaluating use of telehealth with paediatric surgical patients.[16 20] This may be due to the speed with which telehealth was rolled out, with blanket, rather than targeted, use.

It is likely that use of telehealth will continue post-pandemic. Implementation of telehealth long-term will be reliant on development of pathways enabling a balance of face-to-face and remote appointments with an analysis of the impact on care.[21] Bird and colleagues [22] have developed a comprehensive co-design framework to develop virtual clinics for the management of chronic illness in children. Use of such a framework would ensure feelings of disconnect identified in this study are minimised. Service providers must now actively engage with the rapidly evolving technology, such as use of remote stethoscopes [23], understand barriers and support access to technology[21] and develop appointment specific telehealth guidance to optimise use of telehealth.[24]

Parents described the impact that delays and cancellations had to their child's health and developmental progress, with access to community care particularly problematic. A number of parents highlighted the distress caused by feelings of being abandoned by healthcare services. Redeployment had a significant impact on services identified as "non-essential".[25] Our findings mirror

Confidential: For Review Only

those of research with other rare diseases.[26] Continuation of "non-essential" services, such as community Speech and Language Therapy, during periods of high resource would reduce the need for escalation of care to emergency levels, promote development and support parental well-being.

The wider harms caused by society wide "lockdown" have been well summarised[11] and are reflected in our findings; fear and anxiety, displaced non-COVID care, social isolation, stress and loss of income were all reported. Similar themes emerged from research exploring the experiences of parents caring for children with cancer.[27] Our findings highlight the diversity of experiences, typified by divergent management of anxiety that enabled some to transition to a more normal life, while others continued to isolate. Although individual differences in risk evaluation are inevitable, clinicians should acknowledge the burden of decision-making and seek to support by providing clear communication of the best available evidence to mitigate unnecessary isolation.

Parents highlighted the challenge of obtaining information about risks to their child. Not all children were identified as "extremely clinically vulnerable" and advice to shield was mixed. Mixed messages and the need for parents to make their own decisions increased anxiety, also identified in parents of children with cancer.[27] Clinical nurse specialists have been shown to improve patient outcomes,[28] and were identified in this study as excellent sources of advice. The TOFS support group also played an important role in provision of disease-specific information. While individualised assessment may be required, communication between the Government and/or healthcare providers and the charitable sector should be leveraged as a means of sharing accurate and consistent information.

Many parents described the positive impact that isolation had on their child's health and growth. Exposure to common viruses and other infections is usually an inescapable part of childhood and supports development of a well-functioning immune system[29] but can necessitate hospital treatment for vulnerable children. The long-term impact of not being exposed is unknown. However, clinicians should be aware of the potential challenge that some parents will face in supporting normal childhood activity with the knowledge that avoidance may improve health post-pandemic.

Chronic cough is common in children with OA/TOF.[8 30] Interestingly, difficulty differentiating infective coughing from the child's usual cough was not commonly reported and was not hindering school attendance. TOFS provide excellent resources for families to educate about chronic "TOF cough", limiting misunderstanding and empowering parents to advocate appropriately.

Recommendations, based on parental lived experience data, for OA/TOF service providers are presented in table 5. While this study focussed solely on children with OA/TOF it is likely that many of these recommendations would be appropriate for children with other complex healthcare needs.

Table 5. Recommendations for practice

Needs identified	Recommendations for OA/TOF service delivery	Applicable to general service delivery	Applicable to pandemic service delivery
Consistent communication, access to information	Provide a single point of contact within specialist multi-disciplinary specialist services, for example Clinical Nurse Specialist	●	●
	Engage with, and signpost to, third sector organisations to deliver disease specific information	●	●

Confidential: For Review Only

Optimisation of remote healthcare	Use a co-design framework to develop telehealth services to support individualisation of care and meet patient/parent needs	●	●
	Invest in technology to support assessment at home	●	●
Avoidance of harm	Maintain community healthcare services even during periods of high resource need, wherever possible		●
	Acknowledge the burden of parental decision-making during routine follow-up appointments		●
	Identify parental anxiety/mental health concerns related to child's health/development, signposting for appropriate support	●	●

Limitations

Despite efforts to facilitate wider involvement, participants were overwhelmingly female, white parents of pre-school children, likely reflecting those most commonly using Facebook and engaging with a support group. We acknowledge that although a wide range of experiences were described, they may not be reflective of the whole OA/TOF community.

Description of group rather than individual demographics supported anonymity but prevented sub-analysis by OA/TOF type or age. Future research should identify whether such factors impact on satisfaction with telehealth and the assessment of risk.

Conclusion

Parents' experiences of caring for a child with OA/TOF during the pandemic were varied. Rapid adoption of telehealth has demonstrated the enormous potential of remote healthcare delivery but requires refinement to meet the needs of the individual. Future pandemic planning should aim to retain community health care services to avoid escalation of care to an emergency, manage chronic and developmental concerns and support parental well-being. Accurate and consistent, disease-specific information is highly valued by parents. Third sector organisations are ideally positioned to support this.

Confidential: For Review Only

What is already known on this topic

- Direct health risk to children from COVID-19 is low but there is a high risk of "collateral" damage from strategies required to contain the virus.
- Patient support groups can be powerful allies in providing accurate and consistent messages, that is particularly useful to those with rare diseases.
- Social media can facilitate rapid and effective data collection in a rare disease cohort.

What this study adds

- A parent perspective of the impact of pandemic-related reduction in healthcare provision and use of telehealth, highlighting areas of need for pandemic and post-pandemic service delivery.
- An insight into parents' experiences and decision-making surrounding disease containment measures which highlights the variation, even within a single, rare disease.
- Isolation has resulted in exceptionally low exposure to usual childhood infections resulting in improved health for children with OA/TOF.

Confidential: For Review Only

Acknowledgments

The authors wish to thank Joanne Winspear for moderating the Facebook group. Thank you to Diane Stephens, Graham Slater and the team at TOFS for their support and guidance in developing the research and facilitating recruitment. Thank you to Neil Carding, Susan Morris, Hayley Ramm, Julia Faulkner, Kelly Lamour and Helen Marks for their invaluable contributions in the PPI steering group. Finally, thank you to all the parents who gave their time to share their experiences.

Contributorship

AS, JW and CS designed the study. AS led on data collection. All authors were involved in data analysis. AS drafted the manuscript. JW, CS, SE and PDC all revised the work and approved it for submission.

Funding

Alexandra Stewart is currently undertaking a PhD funded by the National Institute for Health Research (NIHR) (award reference: ICA-CDRF-2018-04-ST2-042).

This publication indicates independent research funded by the National Institute for Health Research (NIHR). The views expressed are those of the authors and not necessarily those of the NHS, the NIHR or the Department of Health and Social Care.

Research at Great Ormond Street Hospital for Children NHS Trust is supported by the NIHR GOS/ICH Biomedical Research Centre.

Competing interests

None declared

Exclusive license

BMJ Paediatrics Open adheres to the Budapest Open Access Initiative definition of open access. Articles are published under an exclusive licence or non-exclusive licence for UK Crown employees or where BMJ has agreed CC BY applies. For US Federal Government officers or employees acting as part of their official duties, the terms are as stated in accordance with our licence terms. Authors or their employers retain copyright. Such open access articles can be reused under the terms of the relevant Creative Commons licence to facilitate reuse of the content. please refer to the BMJ Paediatrics Open Author Licence. More information on copyright and authors' rights.

Ethics statement

Approved as part of a larger study by the London Central Research Ethics Committee (20-LO-0098).

Confidential: For Review Only

References

1. Ladhani SN, Amin-Chowdhury Z, Davies HG, et al. COVID-19 in children: analysis of the first pandemic peak in England. *Arch Dis Child* 2020;105(12):1180-85. doi: 10.1136/archdischild-2020-320042 [published Online First: 2020/08/17]
2. Viner RM, Mytton OT, Bonell C, et al. Susceptibility to SARS-CoV-2 Infection Among Children and Adolescents Compared With Adults: A Systematic Review and Meta-analysis. *JAMA Pediatr* 2021;175(2):143-56. doi: 10.1001/jamapediatrics.2020.4573 [published Online First: 2020/09/26]
3. Docherty AB, Harrison EM, Green CA, et al. Features of 20 133 UK patients in hospital with covid-19 using the ISARIC WHO Clinical Characterisation Protocol: prospective observational cohort study. *BMJ* 2020;369:m1985. doi: 10.1136/bmj.m1985 [published Online First: 2020/05/24]
4. Yang J, Zheng Y, Gou X, et al. Prevalence of comorbidities and its effects in patients infected with SARS-CoV-2: a systematic review and meta-analysis. *Int J Infect Dis* 2020;94:91-95. doi: 10.1016/j.ijid.2020.03.017 [published Online First: 2020/03/17]
5. van Lennep M, Singendonk MMJ, Dall'Oglio L, et al. Oesophageal atresia. *Nat Rev Dis Primers* 2019;5(1):26. doi: 10.1038/s41572-019-0077-0 [published Online First: 2019/04/20]
6. Connor MJ, Springford LR, Kapetanakis VV, et al. Esophageal atresia and transitional care--step 1: a systematic review and meta-analysis of the literature to define the prevalence of chronic long-term problems. *Am J Surg* 2015;209(4):747-59. doi: 10.1016/j.amjsurg.2014.09.019 [published Online First: 2015/01/22]
7. Mahoney L, Rosen R. Feeding Difficulties in Children with Esophageal Atresia. *Paediatr Respir Rev* 2016;19:21-7. doi: 10.1016/j.prrv.2015.06.002 [published Online First: 2015/07/15]
8. Kovesi T. Aspiration Risk and Respiratory Complications in Patients with Esophageal Atresia. *Front Pediatr* 2017;5:62. doi: 10.3389/fped.2017.00062 [published Online First: 2017/04/20]
9. Scheider A, Blanc, S., Bonnard, A et al. Results from the French national esophageal atresia register: 1 yr outcome. *Orphanet journal of rare diseases* 2014;9:206.
10. TOFS. 2021 [Available from: https://www.tofs.org.uk/news/2020/03/coronavirus-update.aspx accessed 28th January 2021.
11. Douglas M, Katikireddi SV, Taulbut M, et al. Mitigating the wider health effects of covid-19 pandemic response. *BMJ* 2020;369:m1557. doi: 10.1136/bmj.m1557 [published Online First: 2020/04/29]
12. Neubauer BE, Witkop CT, Varpio L. How phenomenology can help us learn from the experiences of others. *Perspect Med Educ* 2019;8(2):90-97. doi: 10.1007/s40037-019-0509-2 [published Online First: 2019/04/07]
13. Wray J, Brown K, Tregay J, et al. Parents' Experiences of Caring for Their Child at the Time of Discharge After Cardiac Surgery and During the Postdischarge Period: Qualitative Study Using an Online Forum. *Journal of Medical Internet Research* 2018;20(5) doi: 10.2196/jmir.9104
14. Braun V, Clarke V. Using thematic analysis in psychology. *Qualitative research in psychology* 2006;3(2):77-101.
15. Dorsey ER, Topol EJ. State of Telehealth. *N Engl J Med* 2016;375(2):154-61. doi: 10.1056/NEJMra1601705 [published Online First: 2016/07/15]
16. Dean P, O'Donnell M, Zhou L, et al. Improving value and access to specialty medical care for families: a pediatric surgery telehealth program. *Can J Surg* 2019;62(6):436-41. doi: 10.1503/cjs.005918 [published Online First: 2019/11/30]
17. Goedeke J, Ertl A, Zoller D, et al. Telemedicine for pediatric surgical outpatient follow-up: A prospective, randomized single-center trial. *J Pediatr Surg* 2019;54(1):200-07. doi: 10.1016/j.jpedsurg.2018.10.014 [published Online First: 2018/10/23]

Confidential: For Review Only

18. Kemp MT, Liesman DR, Williams AM, et al. Surgery Provider Perceptions on Telehealth Visits During the COVID-19 Pandemic: Room for Improvement. *J Surg Res* 2020;260:300-06. doi: 10.1016/j.jss.2020.11.034 [published Online First: 2020/12/29]
19. Svoboda E, Fruithof J, Widenmann-Grolig A, et al. A patient led, international study of long term outcomes of esophageal atresia: EAT 1. *Journal of Pediatric Surgery* 2018;53(4):610-15.
20. Kohler JE, Falcone RA, Jr., Fallat ME. Rural health, telemedicine and access for pediatric surgery. *Curr Opin Pediatr* 2019;31(3):391-98. doi: 10.1097/MOP.0000000000000763 [published Online First: 2019/05/16]
21. Badawy SM, Radovic A. Digital Approaches to Remote Pediatric Health Care Delivery During the COVID-19 Pandemic: Existing Evidence and a Call for Further Research. *JMIR Pediatr Parent* 2020;3(1):e20049. doi: 10.2196/20049 [published Online First: 25.6.2020]
22. Bird M, McGillion M, Chambers EM, et al. A generative co-design framework for healthcare innovation: development and application of an end-user engagement framework. *Res Involv Engagem* 2021;7(1):12. doi: 10.1186/s40900-021-00252-7 [published Online First: 2021/03/03]
23. Thompson R, Kuryla CL, Kim J, et al. Use of Telemedicine with An Artificial Intelligence-Enabled Stethoscope to Support Decision-Making and Reduce Inappropriate Use of Echocardiography in Children with Heart Murmurs. *Pediatrics* 2021;147(3 MeetingAbstract):989-89. doi: 10.1542/peds.147.3_MeetingAbstract.989
24. Malandraki GA, Arkenberg RH, Mitchell SS, et al. Telehealth for Dysphagia Across the Life Span: Using Contemporary Evidence and Expertise to Guide Clinical Practice During and After COVID-19. *Am J Speech Lang Pathol* 2021;30(2):532-50. doi: 10.1044/2020_AJSLP-20-00252 [published Online First: 2021/02/09]
25. Royal College of Speech and Language Therapists. Redeployment of staff and impact on outcomes for service. London, 2020:1-5.
26. Halley MC, Stanley T, Maturi J, et al. "It seems like COVID-19 now is the only disease present on Earth": living with a rare or undiagnosed disease during the COVID-19 pandemic. *Genet Med* 2021 doi: 10.1038/s41436-020-01069-7 [published Online First: 2021/01/10]
27. Darlington AE, Morgan JE, Wagland R, et al. COVID-19 and children with cancer: Parents' experiences, anxieties and support needs. *Pediatr Blood Cancer* 2021;68(2):e28790. doi: 10.1002/pbc.28790 [published Online First: 2020/11/22]
28. LaSala CA, Connors PM, Pedro JT, et al. The role of the clinical nurse specialist in promoting evidence-based practice and effecting positive patient outcomes. *J Contin Educ Nurs* 2007;38(6):262-70. doi: 10.3928/00220124-20071101-03 [published Online First: 2007/12/07]
29. Ball TM, Castro-Rodriguez JA, Griffith KA, et al. Siblings, day-care attendance, and the risk of asthma and wheezing during childhood. *New England journal of medicine* 2000;343(8):538-43.
30. Sadreameli SC, McGrath-Morrow SA. Respiratory Care of Infants and Children with Congenital Tracheo-Oesophageal Fistula and Oesophageal Atresia. *Paediatr Respir Rev* 2016;17:16-23. doi: 10.1016/j.prrv.2015.02.005 [published Online First: 2015/03/25]

Confidential: For Review Only

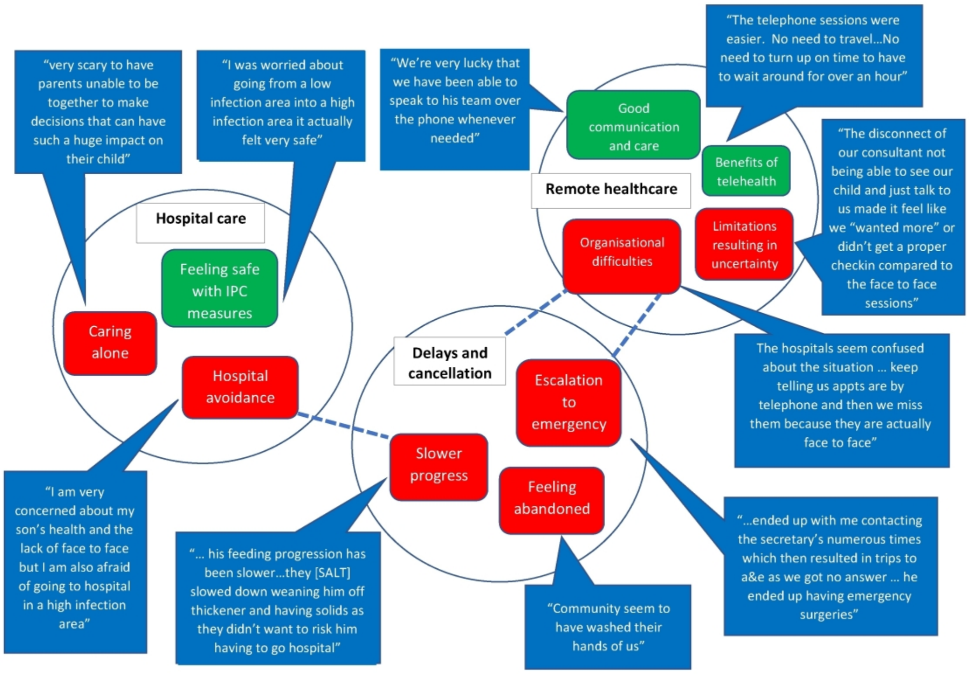

Figure 1: Thematic map healthcare

296x209mm (300 x 300 DPI)

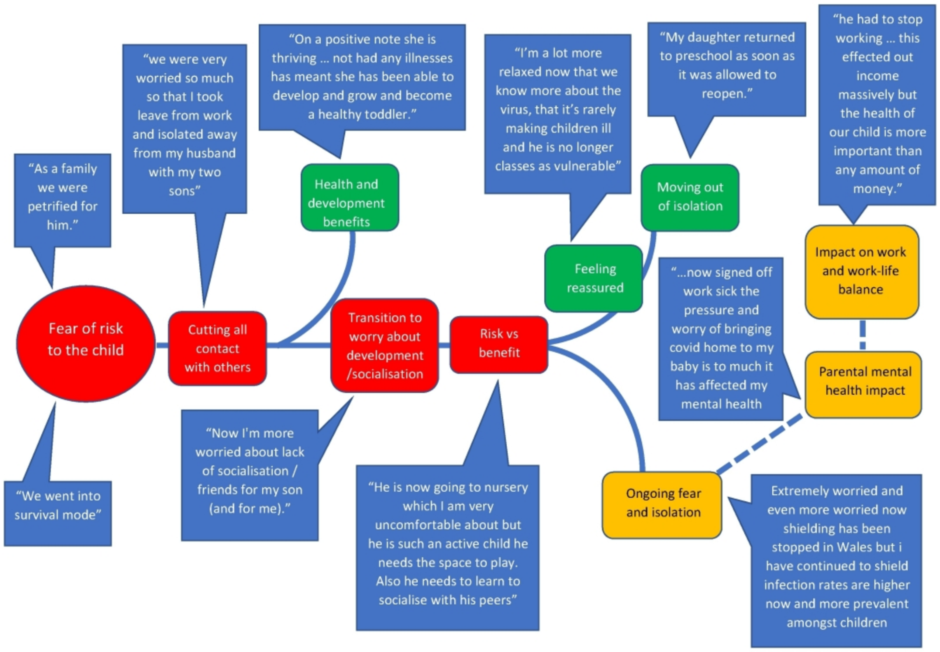

Figure 2: Thematic map non-healthcare

296x209mm (300 x 300 DPI)

Supplementary material

A step-by-step outline of the online forum method

Time point	Task	Action completed by
Pre-recruitment	Write and agree standard operating procedure with support group/charity	Research team<br>PPI group<br>Support group/charity
	Write participant information sheet including statements regarding data use, data storage, confidentiality	Research team<br>PPI group
	Appoint forum moderator	Research team<br>Support group/charity
	Develop questions	Research team<br>PPI group<br>Support group/charity
	Write recruitment advert/publicity material	Research team
	Create Facebook group: set group rules, screening questions for group membership, privacy settings	Research team<br>Forum moderator
Recruitment	Distribute recruitment advert/publicity material (website, email, social media)	Support group/charity
	Interested participants complete screening questions and consent to participation through agreement to group rules	Participants
	Participants meeting criteria for group accepted	Forum moderator
	Participants not meeting criteria for group declined e.g. healthcare professionals	Forum moderator
Data collection	Participants invited to complete demographic questionnaire via link to Survey Monkey	Forum moderator<br>Participants
	First question posted	Forum moderator
	Participants post response to question as a Facebook "comment". Participants engage with other's comments if they wish using "reply"	Participants
	Prompt for more information, further responses or clarification at the moderator's discretion	Forum moderator
	Responses monitored to ensure adherence to group rules. Inappropriate or offensive posts removed	Forum moderator

Confidential: For Review Only

	Further questions posted one at a time, once no further responses to previous question (assumed data saturation)	Forum moderator
Data analysis	Responses copied into a Word document and anonymised	Forum moderator
	Anonymised transcript passed to research team	Forum moderator
	Thematic analysis conducted	Research team
	Analysis triangulated with PPI group	Research team<br>PPI group

Group rules that had to be agreed to before being accepted into the group

**Anonymity**<br><br>I understand that I will provide basic personal information to allow the research team to know who took part in the forum but that they will not be able to identify me by name.
**Information sharing**<br><br>I understand that the results of the study will be published and shared with professionals at conferences and in journals. A summary of the results will be available on the TOFS website and shared in the CHEW newsletter.
**Information sharing (2)**<br><br>I understand that information I give in relation to this project in any online posts may be anonymised and directly quoted in publications and presentations.
**Respect everyone's privacy**<br><br>Being part of this group requires mutual trust. Authentic, expressive discussions make groups great, but may also be sensitive and private. I understand that what's shared in the group should stay in the group.
**Provide information sensitively**<br><br>Please do not use your child's name, or the name of any other individual when you are posting. Please do not post photos or other identifiable content.

Confidential: For Review Only
